# Application of Nanofluids for Machining Processes: A Comprehensive Review

**DOI:** 10.3390/nano12234214

**Published:** 2022-11-27

**Authors:** Aoha Roohi Amin, Ahsan Ali, Hafiz Muhammad Ali

**Affiliations:** 1 Department of Chemical and Energy Engineering, Pak-Austria Fachhochschule, Institute of Applied Sciences and Technology, Mang 22621, Pakistan; 2 Sino-Pak Center for Artificial Intelligence, Pak-Austria Fachhochschule, Institute of Applied Sciences and Technology, Mang 22621, Pakistan; 3 Department of Mechanical Engineering, King Fahd University of Petroleum and Minerals, Dhahran 31261, Saudi Arabia; 4 Interdisciplinary Research Center for Renewable Energy and Power Systems (IRC-REPS), King Fahd University of Petroleum and Minerals, Dhahran 31261, Saudi Arabia

**Keywords:** nanofluids, machining operations, surface roughness, cutting forces, cutting temperature, tool life

## Abstract

According to the demand of the present world, as everything needs to be economically viable and environment-friendly, the same concept applies to machining operations such as drilling, milling, turning, and grinding. As these machining operations require different lubricants, nanofluids are used as lubricants according to the latest technology. This paper compares different nanofluids used in the same machining operations and studies their effects. The variation in the nanofluid is based on the type of the nanoparticle and base fluid used. These nanofluids improve the lubrication and cooling in the machining operations. They also aid in the improvement in the surface roughness, cutting forces, cutting temperature of the workpiece, and tool life in the overall process taking place. It is worth noting that nanofluids are more effective than simple lubricating agents. Even within the nanofluid, the hybrid type is the most dominating, and helps to obtain a maximum efficiency through certain machining processes.

## 1. Introduction

Nanofluids are composed of nanoparticles, which is the latest technology and preferred over common fluids as they are environment friendly. In addition, they have a greater efficiency than most common fluids. In most industrial processes, the primary goal is to efficiently transfer the energy from one point to another by reducing losses. Now, nanofluids are used in different machining operations to minimize these energy losses. The use of different additives (nanoparticles) with the base fluids improves the heat transfer, and so are known as nanofluids. When used for different machining processes, these fluids reflect a higher thermal conductivity than the normal base fluids [1]. Nanofluids have highly enhanced thermophysical properties, including thermal diffusivity, viscosity, heat transfer coefficient, and thermal conductivity. Nanofluids are the colloidal mixtures of the nanoparticles (nano-meter-sized particles) in the base fluids used to improve the heat transfer characteristics suited for the particular application. The nanoparticles used in the nanofluids are mostly made up of metals, oxides, carbides, and carbon nanotubes. They are synthesized by mixing two or more nanomaterials with the base fluids. They are highly effective, and only a tiny amount of the nanoparticles is highly significant in causing a change and obtaining the desired characteristics [2].

In different machining operations, a high amount of heat is generated, which lowers the surface quality of the workpiece and tool life. In addition, it also causes a high amount of driving forces to be acted upon, and, as a result, a large amount of energy, usually in the form of heat, is lost to the environment. This loss is not the only problem in the aspect of energy deficiency. In addition, these thermal losses are not environmentally friendly and damage the environment. Based on the literature, many researchers have tried to find ways to reduce this energy loss so that maximum efficiency can be drawn. High precision can be obtained from the system process. Initially, different cutting fluids were introduced, but these ordinary cutting fluids caused other health and environmental problems. To minimize the effects of these fluids, they were replaced by nanofluids. Due to the perfect features of the nanofluids, they are not only used in various machining operations but also many other industrial applications, such as heat exchangers [3], triangular channels [4], channels equipped with twisted tapes [5], under magnetic fields [6], and many other alike applications. Nanofluids that are composed of nanoparticles and base fluids are preferred over the common lubricating fluids due to them being more effective than the common fluids in almost every viable approach, and the primary purpose of using nanofluids is to increase the overall efficiency by minimizing the losses. Due to advanced research and development, nanofluids are no longer restricted to a single type, i.e., a mono-nanocomposite that is composed of only a single type of nanoparticles, while the approach is extended to the hybrid (binary nanocomposite) and ternary nanofluids (consisting of three composites), where the nanofluids are made from the base fluid and involve more than one type of nanoparticle in its composition.

The properties that affect the pressure drop potentials and aid the heat transfer are generally the thermophysical properties, and, in the case of nanofluids, due to the structural modifications, many of the thermophysical properties are in enhanced forms, such as convective heat transfer, thermal conductivity, viscosity, and the specific heat capacity alike, when compared to the common base fluids [6]. When these common nanofluids give way, they have more enhanced thermophysical properties compared with the hybrid nanofluids, as they reflect better heat transfer properties when compared to the conventional nanofluids. General factors that affect the nanofluids’ thermal conductivity are the shape and size of the nanoparticles, fluid temperature, concentration, thermal conductivity of the nanoparticles, the thermal conductivity of the base fluid, pH value, and other additives [7].

In recent years, the machining performance in various nanofluids has been studied experimentally and numerically. In this article, a general review compares the various effects of the different nanofluids when used for the same machining operation under different and similar conditions. Nanofluids have found applications in different machining operations, where they can enhance the overall efficiency of the operation.

**Figure 1:**
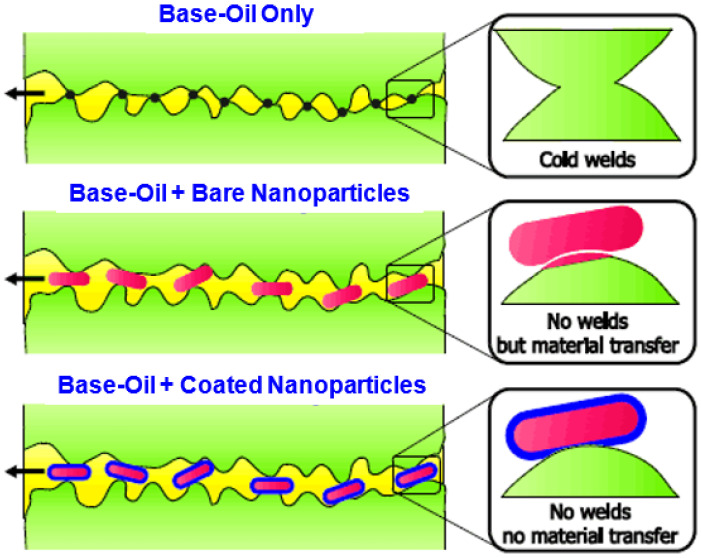
Comparison of the internal phenomenon taking place inside different machining operations under different conditions [8].

Different machining operations have many quality factors considering their lubrication method, such as in Figure 1, where a comparison of the internal phenomenon taking place is shown. In part 1, only the base oil is shown. In part 2, base oil with a bare nanoparticle is shown, and, in part 3, a coated nanoparticle, sometimes also known as a hybrid nanoparticle, is shown, and the impact of each of the three conditions is also shown. This figure depicts that the machining operation in base oil and the hybrid nanoparticle is the most efficient. If the same machining operation occurs in the presence of base oil, it highly damages the overall process and reduces its efficiency.

In the milling machining operation, the latest technology, the nanofluids, is used to solve the problem of the thermal imbalance obtained during the milling process operations. Different researchers have utilized different nanofluids in the milling process and have found different improvements in the overall milling process. These nanofluids improve the surface quality and temperature balance but also help to increase the tool life by avoiding tool wear and tear. Enhanced and high-quality factors help the nanofluids to produce a milling machining operation output with a lower chance of damage and energy loss than the standard milling technique, and highly improve the overall efficiency of the milling machining operations [9].

In the drilling machining operation, the nanofluids that are used for lubrication highly modify the lubricating properties and thus enhance the wettability, enabling the lubrication processes in a much better way and resulting in a reduction in the frictional loss. This reduction in the force of friction is why the cutting forces could be reduced. These nanofluids used in drilling also decrease the surface roughness, i.e., improve the surface quality due to the involvement of the nanoparticles, which increases the heat transfer rate and improves the tool life due to the oil mist and the number of the nanoparticles formed on the flank face. The oil mist formed and the nanoparticles present the possibility of creating a barrier, which causes a reduction in the cutting forces and tool wear. The applications of the nanofluids reduce the tool wear by reducing/removing the heat from the principle shearing zone at a comparatively faster rate and preventing the hardening of the workpiece. Thus, in turn, the cutting tool is sustained for longer and tool wear issues are prevented [10].

Grinding, another machining operation, generally requires a high amount of energy, which produces high temperatures at the workpiece surface as a side effect. This causes damage to the workpiece’s desired properties as it abruptly produces tensile residual stresses, micro-cracks, and surface burns. This increased temperature is controlled by providing improved cooling and lubrication. Thus, different nanofluids are tested in the grinding process, which effectively controls the abnormal thermal effects and overcomes all unwanted effects [11].

In the latest technology of the nanoparticles, it is used as a solution to the problems of the surface roughness and temperature control during turning machining operation. Different researchers have utilized different nanofluids in the turning process and found different improvements in the overall turning process [12]. These nanofluids improve the surface quality and temperature balance but also help to increase the tool life by avoiding wear and tear. Generally, nanofluids are used in various machining operations that help to facilitating the overall process and aid in the performance of the overall process. Within the nanofluids, a major categorization is made according to the primary composition of the nano-additive used in that nanofluid and, based on this idea, nanofluids are classified into three major categories with a variety of sub-categories: carbon-based, metal-based, and composite-based. Nanoparticles generally have modified thermophysical properties that help to produce turning machining operations with a comparatively higher efficiency than the normal turning technique.

## 2. Applications in Various Machining Processes

### 2.1. Nanofluids in Milling

Milling is a machining process that uses a multi-point cutting tool to remove the layer of the workpiece material in the form of grooves from the workpiece surface. It cuts the metal surface as the workpiece is fed against the rotating multipoint cutter. The tool can hold single and multiple cutters with the same data by performing this application [12]. Milling machine operations are of various types. Some of their basic types are plain milling operation, face milling operation, side milling operation, straddle milling operation, angular milling operation, gang milling operation, form milling operation, profile milling operation, end milling operation, saw milling operation, milling keyways, grooves and slots, gear milling, helical milling, cam milling, and thread milling.

Generally, when programming a simple milling machine, different parameters are set for different variables. In order to select these parameters, various factors are kept under observation, including the rigidity of machine tools, workpieces, and cutting tools, the life span of the tool and production rate, the life of the machine tool, the hardness of the workpiece and the heat-balancing conditions, the workpiece perfection and surface quality of the workpiece, and, most importantly, the type of fluid used and cooling method, which highly affects the milling machining operations. Moreover, in this machining operation, controlling and balancing the heat generated is the critical factor to be managed.

In the present scenario, the latest technology of nanofluids is used to solve the problems of thermal balance. Different researchers have utilized different nanofluids in the milling process and found different improvements in the overall milling process. These nanofluids improve the surface quality and temperature balance but also help to increase the tool life by avoiding wear and tear. Nanofluids generally have modified thermophysical properties, such as thermal diffusivity, thermal conductivity, heat transfer coefficients, etc., and these modified properties improve the overall efficiency of the milling machining operation.

The behavior of different nanofluids is discussed in detail in Table 1, where the nanofluids in combination with different nanoparticles are studied from different aspects, a comparison among them is made based on the method of lubrications, workpiece types, and tool types, and various findings are analyzed that are made by several researchers, mainly from the last decade. In most of the studies, it is notable that the minimum quantity lubrication (MQL) approach is used, which is a type of micro-lubrication technique and is preferred as it eliminates large quantities of mineral-oil-based cutting fluids and water and replaces them with a small quantity of environment-friendly lubricants, which are usually nanoparticle-based, most often including vegetable oils mixed with air. In short, we can correctly say that nanofluids help in enhancing the drilling mechanism by the application of the nanoparticles and base fluids that the nanofluid comprises. In addition, different types of carbide tools are mainly used as cutting tool materials due to their specifications, where they easily withstand high temperatures compared to other tool types.

Milling operations are performed on a number of workpiece materials, including titanium aluminum alloy, mild steel, alloys, and different standards of steel, including AISI 1040, AISI 4340, AISI 1018, h-13, Inconel superalloy, Inconel 690, nickel-based super alloys, medium carbon steel, and aerospace alloy.

When carbon-based nanoparticles are used with different base fluids in the milling machining operation, using different references, it has been proven that they highly improve the surface quality by decreasing the surface roughness, such as when using a graphene sheet with vegetable oils, decrease flank wear and central wear, decrease the cutting edges, and enhance thermal conductivity by up to 1.308%. When used with vegetable oil, gold nanoparticles reduce the tool wear, surface roughness, cutting forces, and heat generation xGnP. When used with vegetable oil, they reduce the coefficient of friction, cutting forces, and tool wear by up to 7.45%, and there is a 54.10% improvement in terms of the surface finish. Concerning graphite nanoparticles with MoS_2_, when used with vegetable-based cutting oil, they increase the tool life, decrease the tool wear, and decrease the thermal conductivity by up to 50%. Nanodiamond, when used with vegetable oil, reduces the surface roughness, hBN, MoS_2_, and GnP, and when used with vegetable-based oil, improves the surface roughness, decreases the cutting forces, and improves the surface quality. Carbon onions used with Alumicut oil reduce the cutting forces by up to 21.99%, reduce the surface roughness by 46.32%, and reduce the coefficient of friction. Concerning a graphite nanoplatelet when used with distilled water using 1.6 wt.% conc, the nanoparticles decrease the cutting forces, improve the surface quality, and improve the machining temperature. A nanocarbon onion, when used with mineral oil, reduces the cutting forces by 21.99%, improves the surface quality by 46.32%, and also affects the appearance, function, reliability of the material, hBN, GNP, and SDS, and when used with vegetable oil as a base fluid, it improves the surface roughness and the cutting forces.

**Figure 2:**
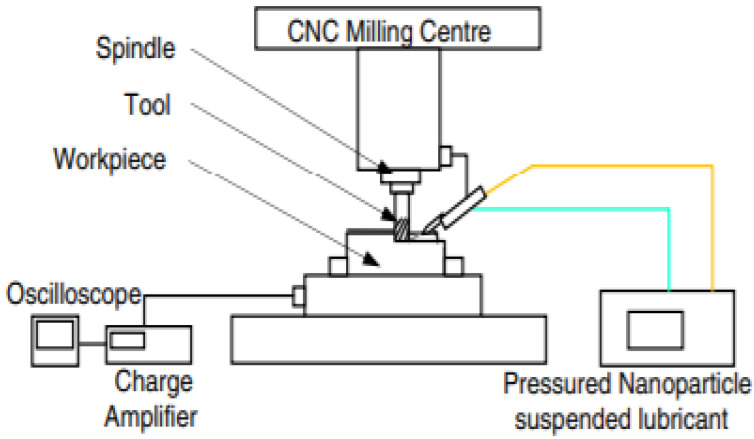
Experimental setup for CNC milling [20].

In Figure 2, the CNC type of milling is taken into account. Its overall experimental setup shows a nanoparticle-suspended lubricant, i.e., the base fluid forms nanofluid, which is used for the surface properties. The overall procedure is similar, with a slight modification, in that the nanofluid is introduced to enhance the overall output of the procedure. These nanoparticles fulfill the requirements of the tool used systematically and have a great positive impact on the workpiece. These nanoparticles are used to improve the overall surface quality just by their thermally stable properties. Due to them, they control the excess release of the heat and maintain the temperature of the workpiece and that of the tool. The machine used is a vertical-type machining center. The cutting speed, feed rate, and depths are selected solely depending on the tool, i.e., as recommended by the tool manufacturer.

The experiment uses a thin-pulsed jet nozzle controlled by a variable speed control drive. The air pressure setting is 50 MPa. AA6061-T6, with a volume of 50 × 50 × 200 mm, is used as a workpiece, the nanofluid is prepared using oil with silicon dioxide nanoparticles, and detailed studies are carried out. After performing the experiment and comparing it with the already existing data, it is concluded that the specific energies, power required at the cutting tool, and cutting forces are considerably reduced using the nanofluids.

**Figure 3:**
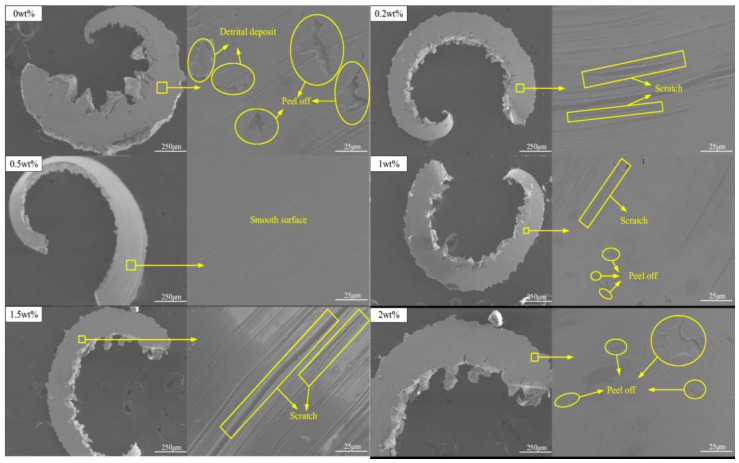
Surface quality for cottonseed oil [21].

Figure 3 shows that a poor surface quality was obtained when cottonseed oil was used following the MQL lubrication technique. The additional scratches found were deep and wide and, when 0.2 wt.% of the nanofluid was used with the same base fluid, the number of scratches was slightly lower than the one with 0 wt.% of the nanofluid. As the nanofluid concentration was increased up to 0.5 wt.%, the overall surface quality was improved. These findings conclude that the concentration of the nanofluid highly affects the overall milling process, and, when including inappropriate amounts, balance the surface quality and other tribological properties.

It is essential to notice that HBN and LN_2_, when used with vegetable oil under the MQL lubrication technique on the tungsten carbide tool, reduce the surface roughness by up to 50%, reduce tool wear, and reduce the cutting forces and heat generation. In addition, they improve the tool life by up to 250%. In comparison, hBN, when used with deionized water under the MQL technique on the tungsten carbide flat end milling cutters, reduces the surface wear by up to 53.89% and helps to provide better lubrication. hBN, MoS_2_ and GnP, when used with vegetable-based oil under the MQL technique on TiAIN-coated carbide, improve the surface quality and decrease the cutting forces.

It is highly significant for the machines to overcome their tearing-like activities by lubrication. Still, according to the Environmental Protection Agency, the conventional methods of lubricating the machinery are damaging the ecosystem and are non-economic. MQL is introduced within the nanofluids in the milling machining process to overcome these hurdles and create environment-friendly techniques. Ref. [36] states that MQL uses a minute amount of the lubrication with a flow rate of approximately 50–500 mL/h only. This, compared to the typical lubrication technique, shows that it is approximately three to four times lower. Thus, we can say that it is more effective.

In the milling machine operations, the comparison studies between the carbon-based, metal-based, and composite-based nanofluids show that the carbon-based nanofluids best serve their purpose in the milling machine operation. This is because the carbon-based nanofluid samples showed a higher thermal conductivity and lower viscosity than the conventional fluids and, within the carbon-based nanofluids, from the table, it is clear that the gold nano-additives-based fluid shows the maximum output with the minimum loss. However, the only shortcoming in using the gold-based nanofluid is that it is not economical. Due to this, it is not generally used. Moreover, a good selection of the type of the nanoparticle-based fluid also depends on the workpiece type. The choice of the type of nanoparticle is also affected by the base fluid and, in the case of carbon-based nanofluids, the most commonly used base fluid is vegetable oil. For the metal-based nanofluids, the most common of the nanoparticles with the most enhanced specifications is molybdenum disulfide used with a variety of base fluids and workpieces, which shows a great decrease in the coefficient of friction, surface roughness, and thermal conductivity. For the case of the composite nanofluids, the basic advantage that they possess is due to different components in the composition of the nanoparticles making a modified and improved version of the nanofluid that generally has enhanced properties required for usage in the milling machine operation [34].

### 2.2. Nanofluids in Drilling

Drilling machining operation forms a hole of different sizes in workpiece materials. In this process, the material is removed, or cutting occurs on the material, in which, the tool uses a drill bit to cut the hole of the circular cross-section in solid materials. Drilling machining operations can be of different types, including plane drilling, core drilling, step drilling, bore drilling, etc. The cutting tool used for the drilling operation is called the drill bit. Drilling is usually performed using a drill machine, which has the advantage of being able to make a hole permanently for a long time. In addition, this machine is needed to mark the end of the components of dresses, especially for setting pockets, darts, etc., whereas, on the other hand, the use of this machine is limited only to drilling purposes.

Factors affecting the drilling machining operation are the type of material being drilled, the rigidity of the drill and the drill press, the rigidity of the work set-up, the quantity/size of the hole to be drilled, and, most importantly, the types and uses of cutting fluid, as its correctness allows for increasing speed, and thus decreasing the time.

Generally, nanofluid is a type of fluid that contains nanometer-sized particles. These are generally sized in the base fluids to form nanoparticle suspension in the base fluid. These nanofluids are mainly used for their enhanced thermal properties, where they act as coolants in the heat transfer equipment. Nanofluids generally have modified thermophysical properties. They actively play a role in improving the drilling machining operations, i.e., due to their thermophysical properties, they have modified properties of thermal diffusivity, thermal conductivity, coefficients of heat transfer, and many more alike. These modifications provide an effective and innovative way to improve their heat transfer characteristics significantly.

When used, nanofluids highly modify the lubricating properties, which improves the wettability and enables the lubrication processes much better, resulting in a minimized frictional loss. This is why the cutting forces could be decreased due to the reduction in the friction force. These nanofluids also reduce the surface roughness, i.e., improve the surface quality due to the involvement of the nanoparticles, which increases the heat transfer rate and prolongs the tool life due to the oil mist and the number of nanoparticles formed on the flank face. The oil mist formed and the nanoparticles present the possibility to create a barrier layer, which suddenly decreases the cutting forces and tool wear. The applications of the nanofluids reduce the tool wear by reducing/removing the heat from the principle shearing zone at a comparatively faster rate and preventing the hardening of the workpiece. Thus, in return, the cutting tool sustains the hardness of the workpiece for longer and prevents tool wear issues.

**Table 2:** Nanofluids in drilling machining operations.

Sr. No.	Nanoparticle Type	Base Fluid	Method of Lubrication	Workpiece Type	Tool Material	Findings/Improvement	References
**Carbon-Based Nanofluids**							
01.	Diamond	Vegetable oil	MQL/nMQL	Ti-6A1-4V	Uncoated carbide twist	Reduces friction Reduces thrust force Reduces drilling torque Decreases tool wear Uses 0.4% wt. conc. of nanoparticle	[37]
03.	Carbon black	Water	HPHT	Aluminum 6061	Carbide tool	Decreases possibility of blocked pipe Fluid loss control ROP enhancement Uses 2 wt.% conc. of nanoparticle	[38]
04.	Graphite alumina	Water-based mud	MQL	Titanium alloy	Carbide tool	Improves effective thermal conductivity Enhances oil recovery Uses 2–4 wt.% conc. of nanoparticle	[39]
05.	Graphene nanosheets	Aqueous solution	MQL	Tool steel	Uncoated cemented carbide tools	Enhance oil recovery Decrease filtrate loss Improve lubricating effects	[40]
06.	Graphene nanosheets and multiwalled carbon nanotubes	Aqueous solution	MQL	Tool steel	Uncoated carbide twist drill	Improve thermal conductivity Improve tribological performance Improve rheological properties Use 3 wt.% conc. of nanoparticles	[41]
07.	Diamond	Paraffin oil	MQL	Aluminum 6061	Uncoated carbide twist drill	Reduces thrust forces Reduces drilling torques Load carrying capacity increased by 5% Coefficient of friction reduced by 15%	[42]
08.	Graphene oxide/phosphorylated graphene oxide	Aqueous solution	MQL	Tool steel	Tungsten carbide flat end milling cutters	Improves thermal conductivity Reduces friction between drill pipe and borehole 9.72% improved energy content of the test fuels	[43]
09.	Multilayer graphene	Aqueous solution	MQL	Titanium alloy	Carbide tool	Enhances oil recovery Improves rheological properties Reduces wear by 75%	[44]
10.	Graphene nanoparticles	Aqueous solution	MQL	Titanium alloy	Carbon steel	Effective chemical inhibition Effective physical plugging Highest shale recovery rate of up to 75.2%	[45]
11.	Carbon black	Water	HTHP/MQL	Fufu-WBM	Carbide tool	Shows significant reduction Filtration properties Reduces water loss by up to 99%	[46]
12.	Multiwalled carbon nanotubes	Aqueous solution	MQL	Carbon steel	Uncoated carbide	Improve energy content of the test fuels Decrease filtrate loss of drilling mud by 6% Use 0.005% *w/v* for the nanoparticles	[47]
13.	Multiwalled carbon nanotubes modified with COOH	Aqueous solution	MQL	Carbon steel	Carbide tool	Decrease filtrate loss of drilling mud Improve energy content of the test fuels Stabilize base fluid thermally	[47]
14.	Multiwalled carbon nanotubes modified with OH	Aqueous solution	MQL	Titanium alloy	Uncoated carbide	Thermally stabilize base fluid by 27% Improve thermal conductivity by 7.2% Improve electrical conductivity by 8.8%	[47]
15.	Cetyltrimethylammonium modified graphene	Aqueous solution	MQL	Aluminum 6061	Uncoated carbide	Improves drilling fluid efficacy Enhances oil recovery Improves operational cost	[48]
16.	Multiwalled carbon nanotubes	Aqueous solution	MQL	Titanium alloy	Carbon steel	Improve thermal conductivity Improve rheological loss Cost-effective	[49]
17.	Carbon-based nanoparticles additives and polymers	Aqueous solution	MQL	Titanium alloy	Carbide tool	Improve drilling fluid efficacy Improve oil recovery Reduce edge radius Improve cooling effect	[50]
18.	Diamond	Vegetable oil	MQL	Ti-6A1-4V	Uncoated carbide twist	Reduces thrust force Reduces drill torque Reduces edge radius Uses 0.2% wt. conc. of nanoparticle	[16,51]
19.	Polymer/graphene oxide composite	Aqueous solution	MQL	Titanium alloy	Carbon steel	Reduces edge radius Improves rheological properties Uses 25% wt. conc. of nanoparticle	[51]
20.	Activated carbon dendrimer incorporating polyvinylpyrrolidone (ACD/PVP)	Aqueous solution	MQL	Titanium alloy	Carbide tool	Recovery capacity of up to 97% Provides super strength Reduces drilling torque	[52]
21.	Nano-diamond	Paraffin oil and vegetable oil	MQL	Aluminum 6061	Uncoated carbide twist drill	Improves cooling effect Improves lubrication Reduces coefficient of friction by 15%	[53]
22.	Graphene-oxide-based novel lubricants (GO, Gly-DES, GO/Gly-DES)	Aqueous solution	MQL	Titanium alloy	Carbide tool	Improve coefficient of drilling fluid by 48.47% Reduce adhesion coefficient of filter cake by 93.33% Use 0.25 wt.% of the nanoparticle conc.	[54]
23.	Multiwalled carbon nanotubes	Aqueous solution	MQL	Titanium alloy	Carbon steel	Improve rheological performance Enhance fluid control loss	[55]
24.	Multiwalled carbon nanotubes	Aqueous solution	MQL	Titanium alloy	Carbide tool	Modify viscosity value Improve shear rate Improve filtrate loss	[56]
25.	Polyacrylamide	Aqueous solution	MQL	Titanium alloy	Uncoated carbide	Improves thermal conductivity Environment-compatible Improves shear stress conc. uses 0.5 wt.% off nanoparticles	[57]
26.	Carbon nanofiber	Water	Cooling	Titanium alloy	Carbide insert	Small cutting temperature Low surface roughness	[58]
27.	Graphene nanoplates	Aqueous solution	MQL	AISI 4140	Carbide tool	Higher thinning properties Lower shear rates Enhanced oil recovery	[59]
28.	Oxidized multi-walled carbon nanotube wrapped by polyethylene glycol	Water-based drilling fluid	MQL	AISI 4140	Carbide insert	Low surface roughness Low cutting temperature	[59]
**Metal-Based Nanofluids**							
29.	Iron	Water	Cooling	Ti-6A1-4V	Carbide tool	Improves cooling action Improves lubrication Is under volume fractions of 1%, 5%, and 10%	[59,60]
30.	Copper	Coconut oil	Dry	AISI 4140	Carbide insert	Decreases flank roughness by 53% Decreases surface roughness by 71% Improves tool life	[56,59]
31.	Zirconium oxide	Aqueous solution	MQL	Titanium alloy	Carbon steel	Avoids filtrate loss Improves operational cost Improves drilling fluids’ efficacy	[59,61]
32.	Titanium dioxide nanohybrids	Aqueous solution	MQL	Titanium alloy	Uncoated carbide	Avoid filtrate loss Improve operational cost Improve drilling fluids’ efficacy	[59,61]
33.	Copper	Soyabean oil	MQL	AA 5052 steel	Carbide insert	Decreases surface roughness by 92% in comparison to the dry drilling	[59,62]
34.	Graphene oxide nanosheets	Aqueous solution	MQL	Titanium alloy	Carbon steel	Improve thermal conductivity Environment-compatible Improve shear stress conc. Use 0.5 wt.% of nanoparticles	[57,59]
35.	Iron	Jatropha oil	Cooling	Ti-6A1-4V	Carbide tool	Improves cooling action Improves lubrication Is under volume fractions of 1%, 5%, and 10%	[59,60]
36.	Silica	Aqueous solution	MQL	Titanium alloy	Carbon steel	Enhances oil recovery Improves rheological properties	[49,59]

It is important to note that most carbon-based nanoparticles enhance the machining process’ overall efficiency when used with different base fluids. Likewise, from “Table 2: Nanofluids in drilling machining operations”, it can be seen that diamond, when used with vegetable oil, reduces the friction, thrust force, tool wear, and drilling torque when used at 0.4 wt.% of the nanoparticle concentration. When used with water, carbon black decreases the possibility of the pipe being blocked and controls the fluid losses. In addition, it enhances ROP when used at two wt.% of the nanoparticle concentration. When used with water-based mud with 2–4 wt.% of the concentration, graphite alumina improves the thermal conductivity and enhances the oil recovery. When used with an aqueous solution, graphene nanosheets improve the oil recovery and decrease the water losses. In addition, they improve the lubricating effects. Using an aqueous solution as the base fluid, graphene nanosheets, and multiwalled tubes with three wt.% concentrations improves the thermal conductivity and tribological and rheological properties. Graphene oxide/phosphorylated graphene oxide used with the same aqueous solution improves the thermal conductivity by 9.72% in the energy content of the test fuels. It improves the friction between the drill pipe and the borehole. In comparison, multiwalled carbon nanotubes at 0.005% of *w/v* concentration decrease the drying mud’s filtrate loss by up to 6% when used with the same aqueous solution as the base fluid. They also improve the energy content of the test fuels. When modified with COOH and used with the same base fluid, these multi-walled carbon tubes decrease the filtrate loss of drilling mud, improve the energy content of the test fuels, and, most importantly, stabilize the base fluid. When modified with OH and used with the same base fluid, these multi-walled carbon tubes stabilize the base fluid by 27%, improving the electrical conductivity by 8.8% and thermal conductivity by 7.2%. When used with paraffin oil as the base fluid, the application of nanofluids in minimum-quantity lubrication grinding increases the load-carrying capacity by 5%, reduces the thrust force and drilling torques, and reduces the coefficient of friction by 15%. When used with an aqueous solution, multilayer graphene improves the rheological properties and wear by 75%. When used with an aqueous solution, it improves the shale rate by up to 75.2% and shows effective chemical inhibition and physical plugging. Similarly, many other carbon-based nanoparticles were studied by several researchers with different and similar base fluids and a characteristic enhancement of the properties was observed in the machining process of drilling. The effects of various nanoparticles include technical and economic benefits. Nanoparticles within the nanofluids improve the rheological properties, thermal and physical occurrences, and effects of the drilling, and reduce fluid loss and mud thickness [63].

One of the recent research projects shows that nanofluids highly influence the drilling machine operations, and they cause a great deal of improvement in this machining process, as they:

○ Reduce the fluid loss, which allows the drilling fluid to perform the drilling uninterrupted;
○ Increase the frequency of the lubrication technique;
○ Reduce the oil in drill cuttings, which effectively helps in the recycling of the base oils in the drilling fluids;
○ Increase the wellbore strengthening.

These are the critical factors observed in the drilling machining procedure irrespective of the type of the base fluid, nanoparticle, lubrication technique, etc.

**Figure 4:**
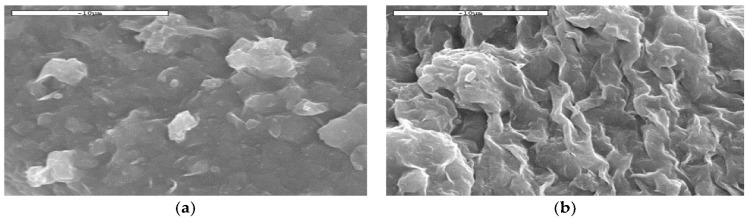
Scanning electron microscope images of the filter cakes formed for the (**a**) base fluid, (**b**) nanofluids [64].

Figure 4 shows scanning electron microscope images of the filter cakes formed after different filtration tests (HP/HT) at pressure conditions of 24.1 bar and 121 °C for the base fluid. They are very smooth without any sort of significant anomalies as shown in Figure 4a, as well as for the nanofluid containing 0.5 wt.% CM Fe_3_O_4_NP, which shows chain-like structures (Figure 4b). Hence, it is concluded from the figure that the presence of these structures improves the surface area of the filter cake, and improves its ability to interact more efficiently and to finally attach firmly to the surface of the filter media.

**Figure 5:**
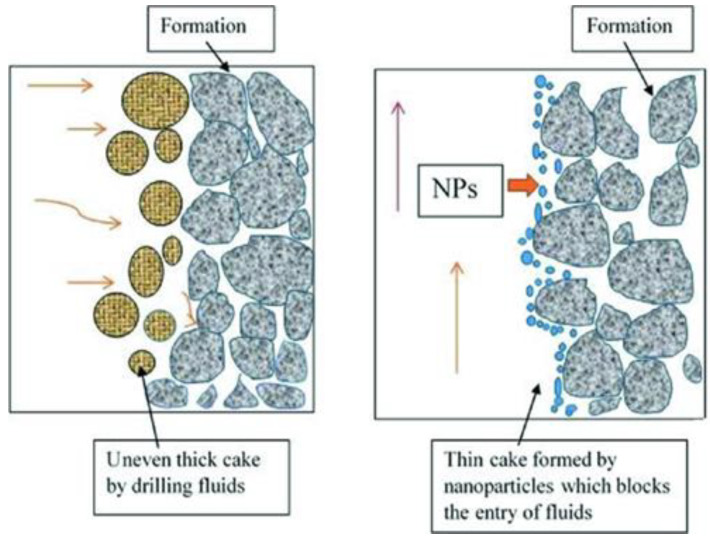
Comparison of the inside of the drilling process [65].

Figure 5 shows a comparison of the inside of the drilling process with and without the drilling nanofluid. In the absence of the nanoparticle, it is visualized that the cake formed by simple drilling fluids is uneven and not uniform. In contrast, in the case of the usage of nanofluids that contain nanoparticles, a thin cake is formed instead of a thick cake. Thus, it serves as a blockage for the entry of the fluids. Therefore, we conclude that the nanofluids help to enhance the drilling fluids by applying the nanofluids. This causes an improvement in the filtration, rheological, wellbore stability, and thermal factors of the drilling mud.

For the drilling machine operations, the comparison studies shown in Table 2 for the different types of nanofluids that vary with the type of nano-additive depict that, of all types of nanofluids, carbon-based nanofluids serve the best in the drilling machines, which is the same as in the milling machine operation. This is due to the uniqueness of the carbon-based nanofluids in the chemical, physical, mechanical, and thermal properties that they utilize to improve the drilling fluids’ efficacy and the operational cost, as, from the table of the usage of the nanofluids in drilling machine operations [46], it is clear that the multiwalled carbon-nanotube-based nano = additives, with a variety of derivatives and with separate based fluids, show a maximum output. This is because they show highly improved rheological and thermophysical properties in this combination, with an increased drilling fluid efficacy [51]. Moreover, a good selection of the type of the nanofluid mainly depends on the workpiece type and the tool material to be used. The choice of the type of nanofluid is also dependent on the nanoparticle combination with the base fluid that best serves its purpose, and the most common base fluids used with the carbon-based nanoparticles are the aqueous solutions that have water content, as their composition also helps in lubricating and obtaining desired phases in order to perform the desired operation. For the metal-based nanofluids, most of the nanofluids based on metal-based nanoparticles are those in which the nanoparticle consists of a nascent metal element, and, in most cases, the metal is not bonded to any other element. In addition, these metal-based nanofluids have a maximized usage of copper and iron with different base fluids, with the most enhanced specifications showing an improved cooling action, lubrication, and oil activity. Although metals generally cause rust, here, the metal-based nanofluids are combined with the base fluids in such a way that they minimize this action [59].

### 2.3. Nanofluids in Grinding

The grinding process is the machining process in which the workpiece is chopped. The rough surface of the workpiece is turned into small and minute-grained particles as required. The grinding machine is also known as a grinder. The grinding process produces particles of very accurate sizes, perfect geometry, and a perfect surface finish. A high-speed specific machining process performs this process, which other simple machining processes cannot perform. A grinding wheel is usually made from thousands of tiny rough/abrasive particles inserted in the matric cells as a bond. In the bonded abrasives, porosity is significant for providing the clearance for the produced chips. and then for providing cooling. Otherwise, chips would interfere with the primary grinding process.

Traditional methods of the grinding process are now undertaken by grinding involving nanofluids; these nanofluids are the emerging technology and significantly play their role in various machining operations. Nanofluid is a fluid containing nanometer-sized particles that are nanoparticles. The nanoparticles used in nanofluids are usually made from carbon nanotubes, carbides, oxides, metal, etc. Nanofluids have perfect properties that make them highly useful in heat transfer and thermal control applications. Due to the factor of perfect thermal properties, they have an improved thermal conductivity and heat transfer coefficient compared to other materials. These qualities make them perfect for the grinding machining process from different aspects.

In the general grinding process, different coolants are used to provide required properties, such as reducing the thermal deformation of the workpiece, avoiding causing wear in the machine, and improving the surface quality. The conventional technique used to fulfill these criteria is hazardous for the environment and living beings. Therefore, this technique has been replaced by the minimum lubrication technique, which enhances previously mentioned characteristics and reduces the cost and environmental impacts [66].

Grinding generally requires high energy, which produces high temperatures at the workpiece surface as a side effect. This may damage the desired properties of the workpiece as it produces tensile residual stresses, micro-cracks, and surface burns. The control of this increased temperature is achieved by providing enhanced cooling and lubrication. Thus, different nanofluids are tested in the grinding process, which effectively controls the abnormal thermal effects and overcomes all unwanted effects.

**Table 3:** Nanofluids in grinding machining operations.

Sr. No.	Nanoparticle Type	Base Fluid	Method of Lubrication	Workpiece Type	Tool Material	Findings/Improvement	References
**Carbon-Based Nanofluids**							
01.	CNT	SAE20W40 oil	Wet	AISI D2 tool steel	Vitrified alumina	Improves surface roughness	[67]
02.	Carbon nanotubes	SAE-20W 40 oil	Wet	AISI D2 tool steel	Silicon material	Surface attributes improve from micro to nano level	[67]
03.	Diamond	Paraffin oil	MQL	SK-41C Tool steel	Vitrified CBN	Improves surface roughness Improves grinding forces	[68]
04.	Diamond	Paraffin oil	MQL	SK-41C tool steel	Vitrified CBN	Decreases surface roughness Decreases grinding force in comparison with the dry and common condition	[68]
05.	Nano-diamond	Paraffin oil	MQL	SK-41C tool steel	Vitrified CBN	Reduces surface roughness Reduces grinding forces	[68]
06.	Diamond	Deionized water	Wet	Al_2_O_3_ grinding wheel	EN-31 steel	Improves grinding temperature Reduce surface roughness	[69]
07.	Diamond	Paraffin oil	MQL	SK-41C tool steel	Al_2_O_3_ grinding wheel	Revealed size, kind, and volume fraction of nanoparticles are pivotal factors affecting the performance of micro-grinding process	[69]
08.	Diamond	Deionized water	MQL	Cast iron	EN-31 steel	Decreases the grinding force Enhances surface roughness Prevents workpiece burning	[70]
09.	ND	Deionized water	MQL	Cast iron	Grinding dish wheel	Yields best G-ratio Produces the best surface when using flood cooling Uses a 4% con. of nanoparticles	[70]
10.	Graphite nanoplates	IPA and TRIM SC200	Wet/Flood	AISI D2 tool steel	Vitrified CBN	Decrease grinding forces Decrease specific energy Enhance surface finish during grinding of hardened D-2 tool steel	[71]
11.	GnP	Vegetable oil	MQL	Tungsten carbide grade YG8	Inconel 718	Reduces grinding forces Reduces friction Improves surface roughness when using MoS_2_ compared to other two	[72]
12.	CNT	Water-soluble oil	Wet and ELID method	Glass	CBN diamond bonded	Surface morphology improvement Surface roughness improvement Micro-crack observation	[72,73]
13.	Carbon nanotubes	SAE-20W 40 oil	Wet	AISI D2 tool steel		Improve surface properties such as micro-cracks and surface roughness	[72,74]
14.	Nanodiamond	Vegetable oil, AF-assisted electrospray	Electrostatic (AF-ESL)	Ti-6A1-4V	Vitrified CBN	Reduces grinding forces Improves workpiece surface Protects the grinding tool Uses 80 nm nanoparticles	[72,75]
**Metal-Based Nanofluids**							
15.	MoS_2_	Vegetable oil	MQL	Tungsten carbide grade YG8	Inconel 718	Reduces grinding forces Reduces friction Improves surface roughness when using MoS_2_ compared to other two	[72]
16.	MoS_2_	Paraffin, CANMIST, and soybeans	MQL	Tungsten carbide	Inconel 718	Decreases tangential grinding force Decreases friction between the workpiece and wear flats Enhances overall grinding performance Improves G-ratio	[76]
17.	MoS_2_	Paraffin, soyabean, CANMIST oils	MQL/Flood	Tungsten carbide	Inconel 718	High G-ratio with MQL lubrication Low G-ratio with flood lubrication	[76]
18.	MoS_2_	Canola oil	MQL	Tungsten carbide	Inconel 718	Produces low grinding force Reduces surface roughness	[77]
19.	Zinc oxide	Water	Nano-coolant or conventional coolant	Ductile cast iron	Al_2_O_3_ grinding wheel	Regulate cutting parameters such as depth of cut, G-ratio, and tool wear	[78]
20.	MoS_2_	Paraffin oil	MQL	Tungsten carbide	Inconel 718	Lowest peak temperature Reduces force ratio Reduces specific energy Decreases surface wear Uses 8% conc. of nanoparticles	[79]
21.	MoS_2_	Paraffin oil	MQL	Tungsten carbide	Inconel 718	Decreases the force ratio and specific energy by up to 45–50%	[79]
22.	MoS_2_	Paraffin and soyabean oils	MQL	Tungsten carbide	Inconel 718	Improves grinding performance Decreases energy consumption Decreases frictional loss Reduces tool wear	[80]
23.	Copper	Water	MQL		Inconel 738 superalloy	Compared to dry lubrication: Enhances surface roughness by 62.16% Improves wheel loading by 59.19% Compared to conventional lubrication: Enhances surface roughness by 36.36% Improves wheel loading by 35.13%	[81]
**Composite-Based Nanofluids**							
24.	Al_2_O_3_	Emulsifier TRIM E709	Wet	EN-31 steel	Al_2_O_3_ grinding wheel	Decreases surface roughness Improves grinding temperature	[69]
25.	Al_2_O_3_	Paraffin oil	MQL	SK-41C tool steel	Vitrified CBN	Improves surface roughness Improves grinding forces	[69]
26.	Alumina	Paraffin oil	MQL	SK-41C tool steel	Al_2_O_3_ grinding wheel	Revealed size, kind, and volume fraction of nanoparticles are pivotal factors affecting the performance of micro-grinding process	[69]
27.	Al_2_O_3_	Deionized water	MQL	Cast iron	EN-31 steel	Decreases the grinding force Enhances surface roughness Prevents workpiece burning	[70]
28.	Al_2_O_3_	Deionized water	MQL	Cast iron	Grinding dish wheel	Yields best G-ratio Produces the best surface when using flood cooling Uses a 4% con. of nanoparticles	[70]
29.	Al_2_O_3_	Water	MQL	AISI 52100	White aluminum oxide grinding wheel	Reduces grinding temperature Reduces surface roughness Improves grinding forces Improves surface morphology	[82]
30.	Al_2_O_3_	Water	MQL	Cast iron	Al_2_O_3_grinding wheel	Decreases force and grinding force Improves ground surface morphology Improves surface roughness when compared to base liquid MQL technique	[82]
31.	Al_2_O_3_	Vegetable oil	MQL	Tungsten carbide grade YG8	Inconel 718	Reduces grinding forces Reduces friction Improves surface roughness when using MoS_2_ compared to other two	[72]
32.	Al_2_O_3_	Water	MQL	Cast iron		Study of the effect of ultra-sonic vibration, concentration of liquid, and pH on the stability of the fluid	[83]
33.	Al_2_O_3_	Water	MQL	Ti-6Al-4V alloy	Sic grinding wheel	Decreases surface roughness Improves grinding temperature	[84]
34.	Al_2_O_3_	Water	MQL	Cast iron	Al_2_O_3_ grinding wheel	Decreases grinding force at small volume fractions	[84]
35.	Al_2_O_3_	Water	MQL	Cast iron	Al_2_O_3_ vgrinding wheel	Lower grinding temperature Lower grinding force Lower roughness of the surface	[77]
36.	Al_2_O_3_	Water	MQL	Hardened AISI 52100	White aluminum oxide grinding wheel	Improves grinding force ratio Reduces surface roughness Reduces grinding forces Improves grinding temperature	[77]
37.	Al_2_O_3_	Deionized water	MQL	Cast iron	Al_2_O_3_ grinding wheel	Produces low grinding force Reduces surface roughness Shows low grinding temperature	[77]
38.	Al_2_O_3_	Water	MQL	Cast iron	Al_2_O_3_ grinding wheel	Key impacts on the nanofluid mist Improves cooling of the grinding region Improves lubrication of grinding region	[73,85]
39.	Al_2_O_3_	Water	MQL	AISI 52100	Ceramic bond aluminum oxide	Increases grinding temperature Heat transfer coefficient analysis	[86]
40.	Al_2_O_3_	Deionized water	Wet	Al_2_O_3_grinding wheel	EN-31 steel	Improves grinding temperature Improves surface roughness	[86]
41.	SiO_2_	Water	Wet	Ductile cast iron	Inconel 718	Measures material removal rate Reduces surface roughness	[87]
42.	Al_2_O_3_, hBN	Water-soluble oil	MQL	Alumina (Purity = 99.8%)	Hard ceramic material	Better cooling performance in the grinding zone at 5% conc. of nanoparticle	[88]

Different nanoparticles, when used with different base fluids, produce enormous effects. These effects are worth noting as they are environment-friendly and constructively affect the workpiece qualities. Considering all of the carbon-based nanoparticles in different forms and used with different base fluids from “Table 3: nanofluids in grinding machining operations”, carbon nanotubes, when used with SAE-20W 40 oil, improve the surface qualities at the nano-level. Diamond, when used with paraffin oil, decreases the surface roughness and grinding force in comparison with the dry and common conditions. Diamond with alumina used with paraffin oil as a base fluid improves the overall performance of the micro-grinding process. When used with the same paraffin oil, it improves the surface roughness and reduces the application of the grinding force. Diamond and aluminum oxide, when used with the same paraffin oil, improve the grinding temperature and surface-roughness-like properties. When used with paraffin oil, nanodiamond reduces the surface roughness and grinding-like properties. Graphite nanoplates as nanoparticles used with IPA and TRIM SC200 as the base fluid reduce the grinding force applications and specific energy required and improve the surface-finish-like surface qualities. Carbon nanotubes with SAE-20W 40 oil improve the surface properties, such as the microcracks and the surface roughness. GnP, aluminum oxide, and MoS_2_ with vegetable oil as the base fluid reduce the grinding forces and frictions and improve the surface quality minutely, but far more than when in the absence of nanoparticles. Nano-diamond that has an 80 nm particle size, when used with vegetable oil and AF-assisted electrospray, reduces the grinding forces, improves the workpiece surface, and, most importantly, protects the grinding tools.

**Figure 6:**
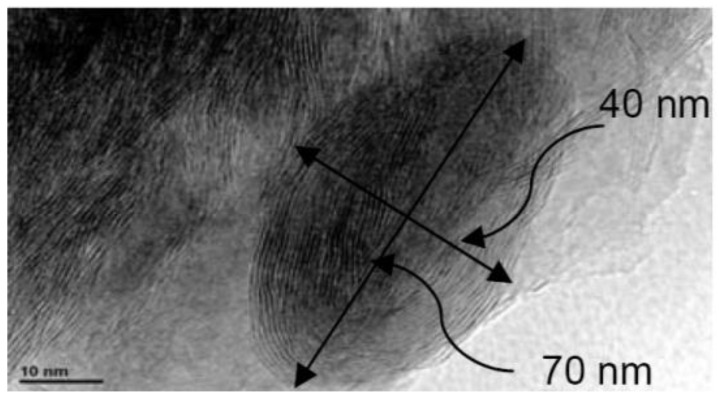
Microscopic view of the molybdenum disulfide nanoparticle [76].

As shown in Figure 6, the molybdenum disulfide nanoparticle is used abundantly as nanofluid in the grinding machining process with different base fluids and produces different enhanced specifications and is thus the most common nanofluid constituent in the grinding machining process.

From one of the references [89], studies show that, in order to reduce the heat generated during the grinding process, a cutting fluid based on nanoparticles, i.e., TRIM E709 with aluminum oxide nanoparticles, effectively reduces the thermal losses in the grinding of the EN-31 steel workpiece. The significant result from this study is that the temperature was reduced by approximately 20–30% compared to the dry and plain emulsifiers, and surface qualities were improved due to the reduction in tool wear. Most importantly, an FEM grinding model was used to stimulate the energy partition, and effectively produced a decrease in the energy partition and surface roughness with the addition of aluminum oxide nano particles.

**Figure 7:**
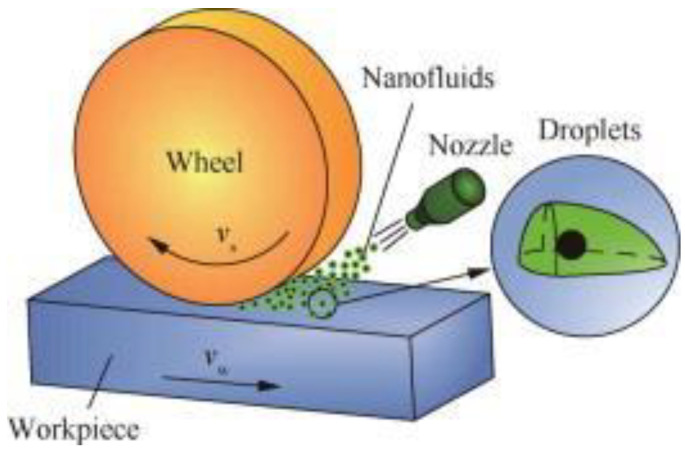
Nanofluid in the grinding machining process [89].

Figure 7 depicts the importance of the nanofluid in the grinding machining process. Whenever nanofluid is used in the grinding process, it highly protects the surface of the workpiece and that of the tool and is regarded as non-hazardous to the environment. This is because it protects the surfaces of the materials involved, which reduces the workpiece’s roughness and helps to increase the tool life at the same time.

In the machine operations that mainly involve grinding, which is somehow relevant to the formation of the granules, the analysis shown in Table 3 for the different types of nanofluids that diverge with the different types of nanoparticles show that, in all of the types of nanofluids used for this machining process, carbon-based nanofluids serve the best as they have the quality of being dominant over all types of nanoparticles in all of the machining operations. This is the case for grinding machining operations, which is mainly because they have a structure that best serves the required function performance; moreover, due to the enhanced thermophysical, chemical, and rheological properties of the carbon-based nanofluids that can be utilized to improve the overall efficiency of the process, especially when multiwalled carbon nanotubes are used in grinding machining operations [89], they help to improve the characteristics from the micro to nano-level, and thus give a good surface finish. This is shown in Table 3, which shows the usage of the nanofluids in grinding machine operations, where it is clear that the carbon-based nano-additives with a variety of separate based fluids reduce the overall grinding forces and improve the overall surface properties, such as surface roughness and micro cracks, by giving the maximum output that they can. More specifically, regarding diamond-based nanofluid with paraffin oil as the most common base fluid, using the MQL lubricating technique improves the overall grinding properties and specifications of the process at the maximum level [74]. Moreover, the perfect choice of the type of the nanofluid (combination of the nanoparticle and base fluid) is mainly affected by the workpiece type and the tool material to be used that best serves the results. For the nanofluids that are categorized into being metal-based, most of the nanofluids based on metal-based nanoparticles are MoS_2_ and, in most cases, variations in the base metal exist with the same nanoparticle, showing the most improved qualities relevant to the grinding process, where they reduce the specific energy by giving an immense advantage over conventional fluids. For the case where some nonmetal or composite element is used as a nano-additive form with any base fluid, it does not serve the process efficiently but still serves the best when compared to common conventional fluids [77].

### 2.4. Nanofluids in Turning

Turning is one of the most common machining operations performed, and usually uses a lathe machine. In this machining operation, excess material is removed from the workpiece to produce the surface of the desired area. A hole may also be made using this process. Turning can be of various types, including plain/straight turning, rough turning, shoulder turning, taper turning, and eccentric turning. There are two types of turning operations, i.e., rough and finish, and each of them differs based on surface properties. The machining process rotates the workpiece, and the cutting tool is transverse along different axes to produce precise diameters and lengths. The bits of the waste metal are known as chips or swarf’s.

The main factors that affect the turning machining operation are the feed rate, cut depth, cutting speed, temperature, and surface roughness. Temperature and surface roughness are the essential key factors because controlling and balancing the heat generated during this process and improving the surface quality to avoid it becoming roughness are the main requirements during this process, alongside the desired function of turning, as these cause not only imbalance effects on the workpiece but also damage the tool.

In this situation, the latest technology of the nanoparticles is used as a solution to the surface roughness and temperature control problems. Different researchers have utilized different nanofluids in the turning process and have found different improvements in the overall turning process. These nanofluids improve the surface quality and temperature balance but also help to increase the tool life by avoiding wear and tear. Nanoparticles generally have modified thermophysical properties, such as thermal diffusivity, thermal conductivity, heat transfer coefficients, etc. They also have an improved viscosity. These factors help to produce a turning machining operation with less chance of damage than the normal turning technique.

The Table 4 shows the study of different nanoparticles using different nanofluids, methods of lubrications, workpiece types, tool types, and various findings made by a number of researchers, mainly from the last decade. In most of the studies, it is notable that the minimum quantity lubrication (MQL) technique is used, which is a type of micro-lubrication technique that is preferred as it eliminates large quantities of mineral-oil-based cutting fluids and water and replaces them with a small quantity of environment-friendly lubricants that usually include vegetable oils mixed with air. In addition, different types of carbide tools are mostly used as cutting tool materials due to their specifications, where they withstand high temperatures easily compared to other tool types.

When carbon-based nanoparticles are used with different base fluids in the turning machining operation, by different references, it has been proven that it highly improves the surface quality by decreasing the surface roughness. MWCNT, when used with coconut oil, improves the surface quality, nano-graphite used with water-soluble oil reduces the surface roughness by 3.1%, carbon nanotubes decrease the surface roughness by 34%, and graphite, when used with water-soluble oil at 2 wt.% conc., provides the best surface quality. When used with vegetable oil, white graphite noticeably improves the surface quality. MWCNT and aluminum oxide, when used with deionized water and vegetable oil, improve the surface quality by reducing the machining force and thus avoiding unwanted heat generation. Gold nanoparticles and aluminum with water and oil acting as the base fluid improve the thermal conductivity and viscosity by up to 3.48% and 17.21% for 0.2 vol.%, 7.44%, and 23.54% for 0.75 vol.%, and 9.03% and 39.24% for 1.20 vol.% concentrations, respectively. GnP, when used with water oil, statistically improves the surface roughness by reducing the tool flank wear by up to 10–15% and improves the tool life by up to 10–15%. In addition, GnP, when used with vegetable oil, improves the surface quality by reducing the tool flank wear by up to 10–15%.

Furthermore, when boric acids vary with the lubrication technique and base fluid, they show a more significant improvement in the specifications, proved by different references: nano-boric acid, when highly used with coconut oil in the wet lubrication technique, causes a decrease in the cutting temperature, surface roughness, and tool wear and tear, and also increases the thermal conductivity and thermal coefficient of heat transfer, thus highly balancing the temperature factor, and, in turn, increasing tool life. When used with SAE40 and coconut oil, nano-boric acid involving the wet lubrication technique reduces the surface roughness, cutting temperature, and flank wear, thus increasing the tool life. Similarly, hBN and aluminum oxide, when used with deionized water and oil with a 0.5 wt.% volume ratio through the MQCL lubrication technique, reduce the surface roughness by 44% of its temperature control property, causing a 37% reduction in the tool wear. Simple hBN, when used with groundnut oil as the base fluid under the MQL lubrication technique, improves MRR by up to 5.09% and reduces the roughness of the surface by up to 28.34%.

**Figure 8:**
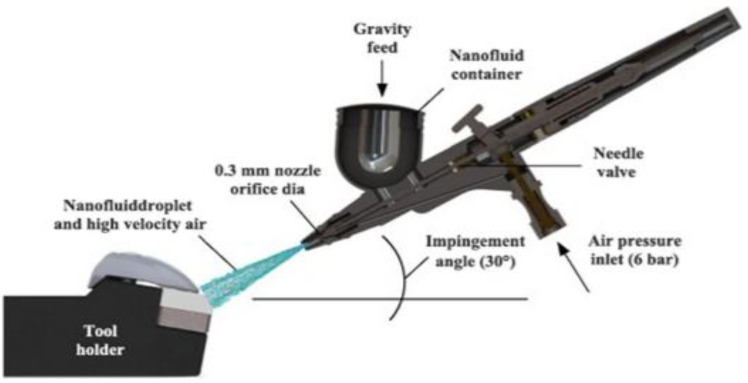
Nanofluid usage in turning machining operation using spraying technique [97].

From Figure 8, the spray cooling technique is the modern method used to reduce the heat generated through the surface of the heated plate. When applied directly in the machining operation, i.e., the turning operation using the spray technique performed by the nanofluids, it causes efficient turning and is a powerful method for reducing the highly heated flux generated at the modified turned surface. Alongside its huge advantage, it still depends on a number of factors, including the nozzle-to-surface distance, nozzle type, heat generation at the surface, type of the nanofluid used, and, finally, the dynamics of the droplets.

In the present studies, simple nanofluids are observed, but hybrid nanofluids (composed of two or more nanofluids) are also critically examined. In one of the articles, published in January 2020, comparison studies of the alumina-based nanofluid were carried out with the hybrid nanofluid of the alumina with multi-walled carbon nanotube nanoparticles, mixed in different concentrations of the volume of 0.25%, 0.75%, and 1.25%, respectively, and various conclusions were drawn by mainly concerning their thermophysical properties.

**Figure 9:**
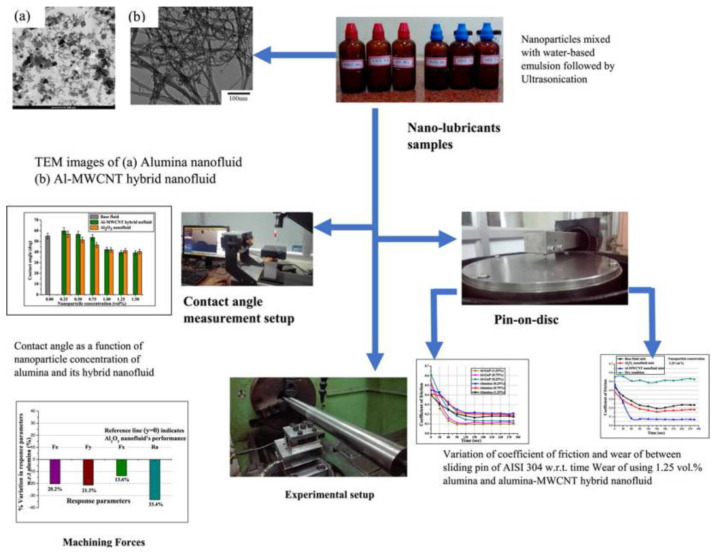
TEM images of different nanoparticles and their effect [108].

As shown in Figure 9, their microscopic structure was first examined under the transmission electron microscope, and changes between the two were observed. These nanoparticles were then separately mixed with the water-based emulsion to form the nanofluid, and different observations of these nanofluids were made, followed by different types of tests. Mainly, the angle measurement and pin on disc test were made for both samples in order to understand the tribological behavior of both nanofluids. The results demonstrated that increasing the concentration of the nanoparticles decreases the wear, so a comparatively low tear was observed in the hybrid nanofluid. In addition, their performances as cutting fluid using the MQL technique were observed by using AISI 304 steel. The result depicted that the performance of the hybrid nanofluid was comparatively better than the alumina-nanoparticles-based fluid.

For the turning machine operations, the analysis from Table 4 for the different types of nanofluids that vary according to the variety of the nano-additives shows that, in all types of nanofluids used for this machining process, the carbon-based nanofluids serve their purpose perfectly as they have the advantage of being dominant over all of the types of nanofluids in all of the machining operations. This is because most of their properties are relevant for the same purpose, and so the same occurs in the case for turning machining operations, which is mainly due to them having properties that serve the best as they help in reducing the cutting temperature, cutting forces, and surface roughness to a great extent. This makes them feasible to use in turning machining operation with the MQL lubrication technique, especially when multiwalled carbon nanotubes are used in the turning machining operation with the water-soluble oil, as they help to improve the overall specifications from the larger scale to the nano scale. Thus, this results in overall improved properties that make their usage advantageous. In addition, it is clear that the carbon-based nano additives vary largely based on the type of base fluids selected and the workpiece and tool type for which the application is desired, which shows that, as a whole, they improve the overall surface properties, such as the surface roughness, by minimizing the cutting forces [109]. Moreover, the correct choice of the type of nanofluid (the combination of the nanoparticle and base fluid) plays a key role when working on the workpiece type and the tool material properly in order to obtain the best results. Furthermore, like the other machining operations, in the case of turning, the metal and composite-based nanofluids are also in use, but they found fewer applications compared to carbon-based nanofluids as they are less advantageous. The most common base fluids with which the latter two types of nano-additives can be used are mainly vegetable oil and deionized or oil-soluble water. The major drawback in the case of using aluminum-based and composite-based nanofluids is that enhancing certain properties tends to decrease the cutting speed, which badly impacts the overall process efficiency [96].

## 3. Impact of Nanofluids in Various Machining Processes

### 3.1. Surface Quality

The surface finish is the surface texture, which is the nature of the surface defined by certain specific parameters/characteristics. This has an essential effect on controlling the friction and wear during the layer-sliding conditions. Surface quality is measured through the surface roughness, lay, and waviness. Surface roughness is the count of total surface irregularities caused after certain processes. Lay is the fibers’ winding direction, i.e., the composition cells’ direction. Waviness is the measure of surface irregularities, with spacing being more significant than that of surface roughness. These characteristics can be measured and then can help to determine the surface properties. Their measurement can be made in two ways, i.e., contact, and non-contact ways, which include various techniques.

Factors that affect the surface roughness or the surface quality are mainly the cutting conditions, tool wear, chip-breaking properties, machine tool, accuracy, and the cutting-edge geometry. These factors highly affect the surface quality. Aside from all of these, lubricating fluid also plays a vital role in improving the surface quality. Different fluids are used to improve this quality, but recent research has proved that using nanofluids significantly increases the surface quality by reducing the surface roughness. During the machining operations, high-energy thermal activities are performed, resulting in the release of a large amount of heat that damages the workpiece’s surface qualities. Nanofluids are used to avoid this damage and modify the surface properties by controlling different parameters. These nanofluids are not only economical but are also environmentally friendly.

**Figure 10:**
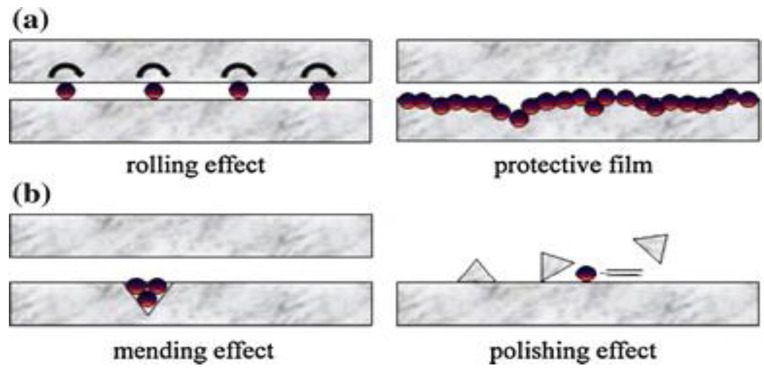
Effect of nanofluid on machining processes (**a**) in rolling and Protective film effect (**b**) in mending and polishing effect [9].

From Figure 10a,b, during the machining operations, when the surface is lubricated with the nanofluid, they create a gentle film on the surface by reducing the chances of tear. This also helps to improve the thermophysical properties of the workpiece, due to which, the heat transfer is controlled, resulting in the safety of the surface by reducing the roughness and improving the quality. The figure shows that whenever the surface gets lubricated with the nanofluid, there is less chance for the surface to get damaged and for the roughness to increase; this all helps to improve the surface quality.

During turning, the highly generated heat flux at the turned surface is controlled by the nanofluids, which, in turn, smoothens the surface of the workpiece and improves its surface properties at the turned face. In contrast, all of the other sides of the workpiece are already uniformly structured. Nanofluids are generally found to have modified thermophysical properties. They help to produce milling machining operations with less chance of damage than the normal milling technique by controlling the abrupt temperature changes that help to produce uniform surfaces by reducing the surface roughness. Due to the nanofluids’ thermophysical properties, they are also effectively used in the process of drilling, i.e., due to these modifications, they help to provide effective and innovative ways to improve the characteristic of abrupt heat transfer during the drilling machining process. Similarly, in grinding, as high energy is provided, which results in significant heat transfer, this heat transfer amount is overcome by the nanofluid. As the heat transfer is reduced, i.e., the temperature is under control, the surface properties are improved.

**Figure 11:**
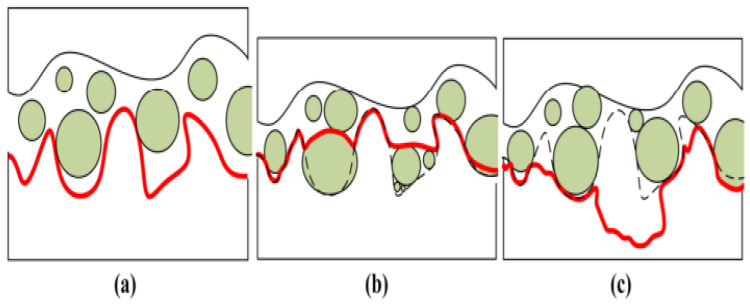
Illustration of the compatibility mechanism for the surface quality (**a**) contact condition of the workpiece, (**b**) lower force and surface roughness from macroscopic view, (**c**) worst surface quality where wear is greater than the polishing effect [116].

In Figure 11a, the contact condition of the workpiece is shown, where different conditions are described under different observation angles and compatibility mechanism profiles are shown with red-color curve. The boundary formed by the fluid is uncertain under the actual conditions, where the plastic deformation occurs for the nanoparticles, but, in this case, the overall friction gets reduced. At the same time, along with the deposition at the crusts, some of the nanoparticles get deposited at the troughs, which reflects the lower force and surface roughness as shown in Figure 11b. When these particles get into troughs, they cause surface wear by breaking away the debris. In addition, From Figure 11c, it is noticeable that the surface quality gets worse whenever the wear is more significant than the polishing effect. It is highly recommended to use nanofluids that improve the surface quality by reducing the wear and roughness-like properties in all of these conditions.

When the base fluid is used with the nanoparticles in the milling process, it usually decreases the surface roughness by improving the surface quality. GnP with vegetable oil [13], nano diamond with vegetable oil [16], carbon onions with Alumicut oil, nanocarbon onions with mineral oil [17], graphite nanoplatelet with distilled water [18], GNP with vegetable-based oil [19], HBN with vegetable oil, Al_2_O_3_ with water [33], MoS_2_ with ECOCUT HSG 9055 oil, TiO_2_ with water, MMT clay with water oil, montmorillonite clay with water-soluble mineral oil [26], copper nanoparticle with water-soluble oil, TiO_2_ with lubricant emulsion [30], copper with oil [31], ZnO with EG [32], Al_2_O_3_ with palm oil [33] and hBN with vegetable-based oil [34] when used as nanofluid in the milling machining process, all show a significant improvement in the surface quality of the workpiece by the reduction in surface roughness properties.

In the process of drilling machining, graphene nanosheets with an aqueous solution, multiwalled carbon nanotubes with an aqueous solution [40], multilayer graphene with an aqueous solution [44], silica with an aqueous solution [49], polymer with an aqueous solution, graphene oxide composites with an aqueous solution [51], copper with coconut oil [56], copper with soyabean oil [62], carbon nanofibers with water [58], and oxidized multi-walled carbon nanotubes wrapped by polyethylene glycol with water-based drilling fluid [59] all show a significant reduction in the surface roughness by the improvement in the surface quality.

In the process of grinding, Al_2_O_3_ with emulsifier trim E709, CNT with SAE20W40 oil, diamond with paraffin oil, Al_2_O_3_ with water, diamond with paraffin oil, CNT with water-soluble oil, nano-diamond with paraffin oil, graphite nanoplates with IPA and TRIM SC200, GnP with vegetable oil, Al_2_O_3_ with vegetable oil, MoS_2_ with vegetable oil, nano-diamond with deionized water, and nano-diamond with vegetable oil, when used, all improve the surface properties in the noticeable parameters, which causes an improvement in the surface properties and lessens the surface roughness. The comparison studies of different types of nanofluids in various machining operations show that carbon-based nanofluids generally best serve all machining processes as they have enhanced heat transfer properties, and that different derivatives of carbon-based nano-additives, when used with a variety of base fluids, best serve their purpose, and help to maintain the surface quality of the workpiece, as well as of the tool, by minimizing the overall surface roughness and friction coefficient.

When the base fluid is used with the nanoparticles in the turning process, it usually decreases the surface roughness by improving the surface quality. graphite with water-soluble oil [91], boric acid solid lubricant with coconut oil, white graphite with vegetable-based oil [92], MWCNT with deionized water and vegetable oil [108], GnP with vegetable oil [94], Nano-boric acid used with coconut oil [117], nano-boric acid with SAE40 oil [109], hBN with groundnut oil [110], MWCNT used with coconut oil, nano-graphite used with water-soluble oil, CNT, silver nitrate used with sodium borohydride [99], Cuo used with water-soluble oil [101], Al_2_O_3_ with Servo-cut ‘S’ and with vegetable oil [112], hBN with DW-oil [114], ZnO with deionized water, CuO with deionized water [104], and Cu with vegetable oil [106] all show an incredible reduction in the surface roughness by improving the surface quality.

### 3.2. Tool Life

Tool life concerns how long certain tools can typically function, i.e., the total time for which the same tool can be used in certain machining operations. It is a common understanding that the more the tool is handled with care, the more likely it can be used in the long term, and vice versa. Traditional machining operations are highly unfriendly as they not only cause damage to the workpiece but also damage the tool, as these methods release a large amount of heat, which is uncontrollable and results in the production of unwanted effects caused either on the tool or on the workpiece.

Using technological development in nanoparticles, nanofluids can be used as lubricating agents in machining operations, which are very effective in terms of the economy and environment. These nanofluids cause an improvement in the rheological and thermophysical properties of the materials, either in the form of the workpiece properties or the properties of the tool used in that particular machining process.

Nanofluids, when used as lubricating agents in the turning process, improve the tool life as less contact force is required to make the change in the workpiece using the tool, due to which, less impact is needed. This, in turn, reduces the overall function of the tool, which, in turn, improves the tool life. Similarly, in the other machining operations, lubrication helps to create changes quickly, and these changes can reduce the overall functionality of the tool, which helps to increase the tool life by reducing wear [118].

**Figure 12:**
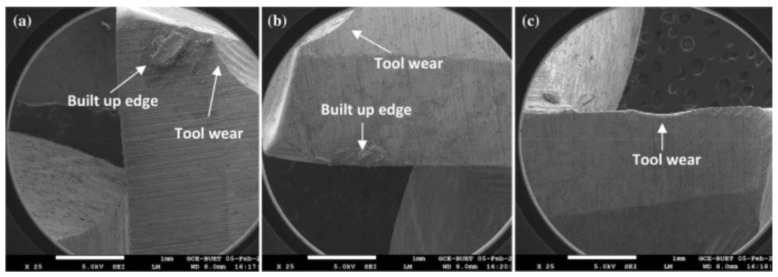
Effect of machining operations performed under different conditions (**a**) in dry, (**b**) with conventional cutting fluid, (**c**) with nanofluid [119].

In Figure 12, the results are compared when machining operations are performed under different conditions. In Figure 12a, the cutting operation is performed in the dry lubrication technique, in Figure 12b, the conventional fluid is used for the lubrication, and Figure 12c involves the nanofluid for the lubrication. It is depicted that tool wear is significantly reduced in the case of nanofluid, due to which, there is less chance of damage to the fluid, which indicates that the tool life will be improved so far in this case when nanoparticles are used with a certain base fluid in the machining process. This figure is deduced explicitly after any particular machining process, but the same concept applies for all the other machining operations when carried out by using different nanofluids for different operations. Their selection is based on their quality in terms of specific parameters required in that particular operation.

In the process of milling, graphene sheets with vegetable oil [12], MoS_2_ with vegetable-based oil, GnP with vegetable-based oil [13,14], graphite nanoplatelets with distilled water [18], HBN with vegetable oil [19], LN_2_ with vegetable oil [120], silicon dioxide with water [23], montmorillonite clay with water-soluble mineral oil [26], silicon dioxide with mineral oil [35], silver with EG, ZnO with EG [32], Al_2_O_3_ with palm oil [33] and hBN with vegetable oil [34], all reduce the machinery wear and tear, and thus the machine damage is reduced, which, in turn, improves the machine life time, and so improves the tool life specifically.

In the process of drilling, the life of the tool is improved by a reduction in the wear and tear caused between the tool and the workpiece material. Due to this, the improved quality of the tool is maintained for longer, and the same applies for the workpiece, where its quality damage is reduced, and less flanks and damage exist on the surface of the workpiece. In this machining process specifically, diamond with vegetable oil [37], multilayer graphene with an aqueous solution [44], iron with water [60], graphene nanosheets with an aqueous solution, multiwalled carbon nanotubes with an aqueous solution, multiwalled carbon nanotubes when modified by the addition of OH and with an aqueous solution, multiwalled carbon nanotubes when modified by the addition of COOH and with an aqueous solution, ND with paraffin oil, ND with vegetable oil [53], zirconium oxide with an aqueous solution, titanium dioxide nanohybrids with an aqueous solution [61], graphene oxide nanosheets with an aqueous solution [57], carbon nanofiber with water [58], graphene nanoplatelets with an aqueous solution [59], and copper with coconut oil all significantly improve the tool life.

During the grinding machining operation, the tool piece acting as a grinder has the highest chance of damage and decreasing the tool life as they have to apply shear forces on the workpiece, although the tool in this case gets damaged at the most rapid rate compared to the other machining operations. Although it is difficult to control the tool damage, it is not impossible, and this damage can be controlled comparatively. In addition, the tool life can also be increased when compared to the traditional grinding process and its technique. This positive change, i.e., the improvement in the tool life, can be achieved by using nanofluids, i.e., nanoparticles with some base fluid as a lubricating agent, instead of the conventional lubricants, which are non-economic as well as environmentally damaging.

In the process of turning, carbon nanotubes, graphite with water-soluble oil [91], GnP with vegetable oil [94,121], nano-boric acid with SAE40 oil [109], Al_2_O_3_ with deionized oil, hBN with DW-oil [110], AgNO_3_ with sodium borohydride [99], CuO [101], Al_2_O_3_ with vegetable oil [102], silicon dioxide with mineral oil, boric acid solid lubricant with SAE-40 coconut oil, white graphite with vegetable-based oil, copper with grease, Al_2_O_3_ with Servo-Cut-‘S’ [112], Al_2_O_3_ with DW-water [104], ZnO with deionized water, CuO with deionized water, and GnP with water oil [107,115] all significantly reduce the tool wear and thus improve the tool life, and so the same tool can be used for longer in the turning machining operation.

Due to the enhanced thermophysical properties offered by the carbon-based nanofluids, they help to provide the best surface quality, along with good mechanical properties, which help in maintaining the mechanical prospective of the machining operations as well. This, in turn, helps to maintain the tool’s good overall performance, maximizing the tool life by minimizing the damages to it, where the quality of having an improved surface quality is interrelated with maintaining the tool and the workpiece in a stable condition.

### 3.3. Cutting Forces

Cutting forces are actually the driving force for certain machining operations. These forces have a great impact on the tool, as well as on the workpiece in terms of the tool life, workpiece surface properties, and other similar characteristics. The magnitude of the cutting forces is responsible for the strength of the machining operation that is taking place. These cutting forces help the machining operation to perform certain tasks. The greater the cutting force, the less time required to perform certain machining operations but, at the same time, these forces cause damage to the workpiece and the tool, as a greater force has a bad impact on them, i.e., the greater the force, the greater the chances of cracks on the surface, and so greater the chance of causing surface roughness of the workpiece. In a similar way, its greater magnitude also causes tool wear, which damages the tool quality and reduces the tool life. Conventional ways of machining operations require greater cutting forces, and the only advantage that these greater cutting forces have is that they save time.

**Figure 13:**
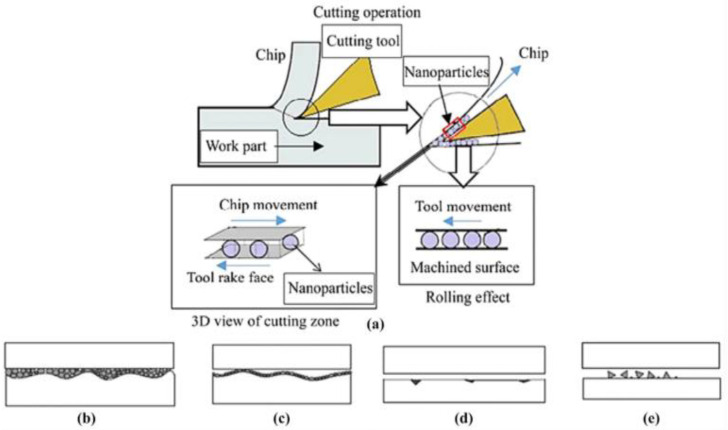
Cutting machining operation (**a**) simple cutting operation, Effects of nanofluids (**b**) rolling, (**c**) protective film, (**d**) mending and (**e**) polishing [120].

In Figure 13a, the simple cutting is shown: whenever any force is applied on the workpiece through the tool, the required result is obtained. When these are lubricated with the nanofluids, the change is made easily and, at that point, a lower amount of energy is required, i.e., comparatively less cutting force is to be applied when nanofluid is used. In Figure 13b–e, the rolling effect, protective film, mending effect, and polishing effect are, respectively, shown when nanoparticles are used with certain base fluids, which helps to increase the overall efficiency of the particular machining operation.

**Figure 14:**
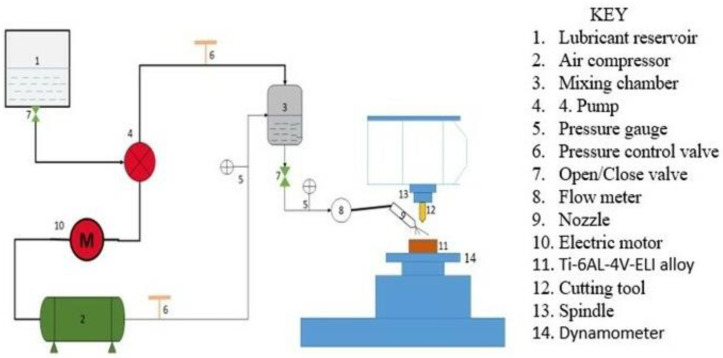
Experimental setup for machining process [122].

Figure 14 shows the overall experimental setup for the machining processes and clearly reflects that the cutting forces are to be applied by the tool on the workpiece, and that these cutting forces must be applied in a significant amount so that they can cause the performance of the desired machining operation.

The technological advancements have developed nanoparticles that are highly significant in terms of machining operations, i.e., these machining operations involve different lubrication techniques and, when the lubrication is carried out using nanoparticles with the base fluids, the overall performance of the machining operation is increased. When nanofluids are applied for any machining operation, a gentle surface is created, due to which, less force is required for any operation, and so the same result can be obtained with a reduced application of the cutting force. As the cutting force gets reduced, a lower amount of the driving force is needed to be applied for performing the particular operation. This not only reduces the amount of the force that is to be applied, but also reduces the chances of cracks on the surface of the workpiece, i.e., reduces the surface roughness by improving the surface quality overall, which involves different parameters for its measurements. In addition, this reduced force application helps to apply comparatively less force through the tool on the workpiece, so there is less chance of damage to the tool, i.e., even if the wear at certain angles does occur, it is actually at a lower ratio than before. This helps to reduce the wear and tear of the tool and so increases the tool life. Thus, in short, we can say that, by using nanofluid, the cutting forces are reduced, which leads to an improved surface quality and increased tool life.

In the milling machining operation, different nanofluids show a noticeable reduction in the magnitude of the cutting forces, which helps to obtain the maximum benefit out of the operation, in such a way that the amount of force applied is reduced, and then all of the work carried out is converted to a useful trait and the losses due to heat and other changes are minimized. A few of the possible most important combinations of the nanoparticles with the base fluids are GnP with vegetable oil [13], hBN with vegetable-based oil, MoS_2_ with vegetable-based oil, carbon onions with Alumicut oil [17], graphite nanoplatelets with distilled water [18], GO with metal work coolant, nanocarbon onions with mineral oil, silicon dioxide with ECOCUT SSN 322 mineral oil [20], Fe_3_O_4_ with water and oil [21], LN_2_ with vegetable oil [120], HBN with vegetable oil, MoS_2_ with deionized water [22], TiO_2_, with water [23], MoS_2_ with ECOCUT HSG 9055 oil [24], MoS_2_ with ECOCUT HSG 905S neat cutting oil [25], MMT clay with water oil [26], silicon dioxide with mineral oil [35], Al_2_O_3_ with oil [31,33], Ag with EG [32], ZnO with EG [32], and Al_2_O_3_ with palm oil [33,34], which all show a significant decrease in terms of the cutting force when used at a nanoparticle size.

In the process of drilling, a large amount of force is required in order to satisfy the requirement of the workpiece so that the desired function can be performed accurately at the accurate points. This can be carried out when a large amount of force is applied in such a way that it does not disturb the morphology of the workpiece, as well as that of the tool. When diamond with vegetable oil [38], graphene nanosheets with an aqueous solution [41], multiwalled carbon nanotubes with an aqueous solution [42], diamond with paraffin oil [43], multilayer graphene with an aqueous solution [45], cetyltrimethylammonium-modified graphene with an aqueous solution [53], activated carbon dendeimer incorporated by polyvinylpyrrolidone (ACD/PVP) with an aqueous solution [57], graphene-oxide-based novel lubricants (GO, Gly-DES, GO/Gly-DES) with an aqueous solution [59], oxidized multi-walled carbon nanotubes wrapped by polyethylene glycol with water-based drilling fluid [66] and graphene nanoplates with an aqueous solution are used in such a way that the nanoparticles are combined with the base fluid so that they are used as a lubricating agent in the drilling machining operation, the overall efficiency of the drilling machine process is improved.

In the grinding machining process, there is a maximum count for the cutting force that is to be applied in order to obtain the outcome required, as the machining process is required to be performed. In the grinding, larger cutting forces are required regardless of the technique used. This cutting force also results in greater thermal losses that need to be overcome; otherwise, they can damage the quality of the workpiece, as well as that of the machinery and its tool. Avoiding thermal losses is actually not possible as no part of the system can generate a 100% efficient output; thus, we can rephrase our statement, where, in order to reduce thermal losses, a nanofluid lubricating technique is used in the grinding machining operation as well, which helps to minimize the losses. In this way, the cutting force applied is converted to the maximum output. When diamond is used with paraffin oil [69], Al_2_O_3_ is used with water, Al_2_O_3_ with paraffin oil [70], graphite nanoplates with IPA and TRIM SC200 [72], GnP with vegetable oil [74], and MoS_2_ with vegetable oil [74], MoS_2_ with paraffin oil [75], MoS_2_ with CANMIST [75], MoS_2_ with soyabean oil [75], nanodiamond with deionized water [90], such that nanoparticles, with the base fluid as a whole, form nanofluids that reduce the thermal losses, the cutting forces are minimized such that all of the forces applied get converted into useful work carried out.

In the turning operation, various nanoparticles combined with various base fluids show an observable reduction in the cutting forces, which helps to improve the overall efficiency and performance of the turning machining operations. A few of the most important combinations of the nanoparticles with the base fluids include nano-graphite with water-soluble oil [91], graphite with water-soluble oil [92], GnP with vegetable oil [97], nano-boric acid with coconut oil [98,99], MWCNT [102], which reduces the cutting temperature by up to 65%, CNT, which reduces the cutting temperature by up to 33%, AgNO_3_ with sodium borohydride [104], CuO with water-soluble oil [106], Al_2_O_3_ with Servo-Cut-‘S’ [110], Al_2_O_3_ with deionized water [113], CuO with deionized water [113], MoS_2_ with coconut oil [118], etc. All of these combinations show a significant reduction in the cutting forces when the material is used in the form of the nanoparticle.

Carbon-based nanofluids have a higher temperature-dependent thermal conductivity and enhanced heat transfer coefficient at generally low concentrations, which make them the best choice for their selection in different machining operations in order to provide a reduction in the cutting forces so that the desired operation is performed perfectly with a generally good efficiency.

### 3.4. Cutting Temperature

As in the machining process, the ultimate aim is to change the morphology of the workpiece into the desired shape by using a tool. These machining processes also require proper lubrication, which, if not performed, causes wear and tear in the machine. As a result, the desired intensity of the performance of certain machining operations may not be obtained. If the machining operation lacks lubrication, it causes damage to the surface of the workpiece in the form of surface roughness by reducing surface qualities, as it causes wear and tear in the machines. Therefore, there is a threat toward the damage of the tool’s life span. Moreover, cutting forces are required at a greater magnitude in the proper direction because a greater amount of energy is required to create some change in the workpiece surface. The only way for cutting forces to be reduced is to increase the surface temperature, i.e., more often, the cutting temperature. When the temperature of a certain body is increased, it is easier to mold its shape and transform it into some other shape.

In the traditional ways of performing the machining operations, a greater cutting temperature is utilized, not for reducing the cutting forces, but because some of the temperature is provided to facilitate the cutting whereas the rest of the thermal energy is due to the heat transfer between the tool and the workpiece.

Regarding advancements, nanotechnology has created ease in the modern world and facilitates us in almost each and every sector of life. Similarly, in the machining operations, when nanofluids (nanoparticles homogenized with the base fluids) are used for the lubrication of machines, this causes flexibility in the conditions that are to be provided while performing any sort of operation. When nanofluids are used, they form a gentle and smooth processing by facilitations in such a way that a lower cutting force is required comparatively, as less energy is required to create change in the workpiece surface. This causes the surface morphology to be protected, which also enhances the surface qualities, which is the result of a reduction in the surface roughness. In addition, this also causes a reduction in the rate of the wear and tear caused by the machine, due to which, the tool gets protected, and the tool life is enhanced.

**Figure 15:**
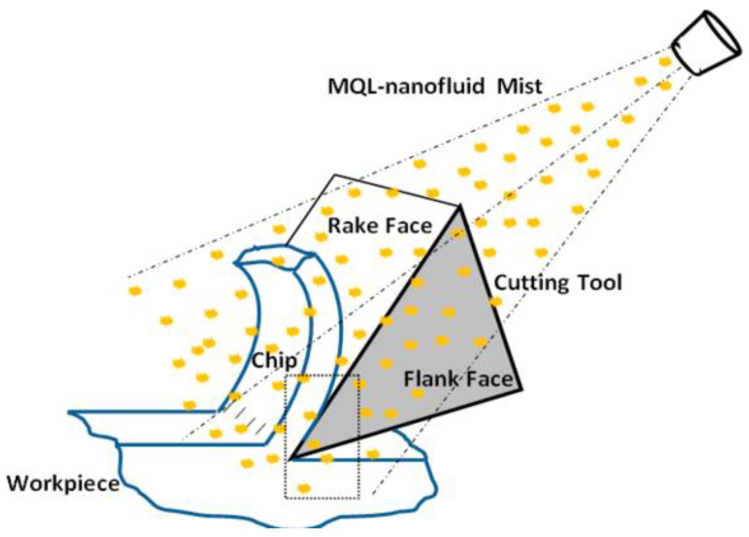
Effect of nanofluid on machining operation [123].

In Figure 15, it is shown that whenever nanofluid mist is passed through the tool in a certain machining process, it performs the operation accurately and, in a time, -efficient manner, i.e., the tool fits smoothly to the workpiece and performs the desired operation, thus reducing the magnitude of the cutting forces that are to be applied for the operation.

When graphene sheets are used with vegetable oil [12], GnP with vegetable oil [13], graphite nanoparticles with vegetable-based cutting oils [14], GO with metalwork coolant [17], graphite nanoplatelet with distilled water [18], silicon dioxide with ECOCUT SNN 322 mineral oil [20], MoS_2_ with deionized water [22], Al_2_O_3_ is used with water [23], TiO_2_ with water [23], MoS_2_ with ECOCUT HSG 9055 Oil [24], MoS_2_ with ECOCUT HSG 9055 neat cutting oil [25], MoS_2_ with vegetable-based cutting oil [26], silicon dioxide with mineral oil [35], silver with EG [32] and zinc oxide with EG [32] all of these nanoparticles combined with the base fluid that form the nanofluid significantly reduce the cutting temperature in the milling machining operations.

**Figure 16:**
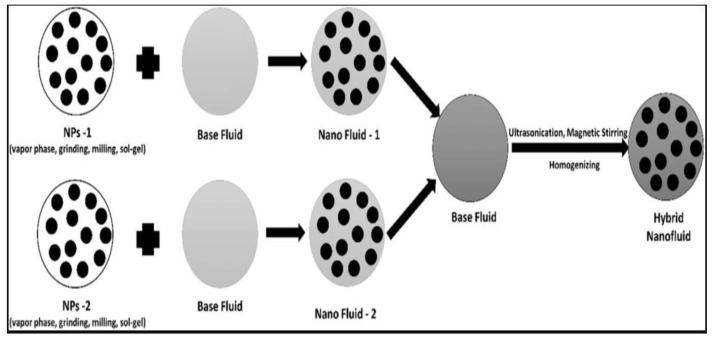
Hybrid nanofluids as renewable and sustainable colloidal suspensions for potential photovoltaic/thermal and solar energy applications.

As shown in Figure 16, in order to reduce the cutting temperature, hybrid nanofluids are mostly used instead of single composition nanofluids. These nanofluids are formed in the same way, except that those two types of nanoparticles are used with the base fluids. In the figure, the composition of the hybrid nanofluid is shown, where two different nanoparticles are combined with different or possibly similar base fluids that are then combined in another base fluid. Then, after their proper homogenization, they are turned into hybrid nanofluids, which are then used for various purposes and, more specifically, for lubrication purposes in the machining operations.

In the drilling machining operations, graphene nanosheets with an aqueous solution [40], multiwalled carbon nanotubes with an aqueous solution [41], graphene oxide/phosphorylated graphene oxide with an aqueous solution [43], iron with water [60], multiwalled carbon nanotubes modified with OH with an aqueous solution, graphene-oxide-based novel lubricants (GO, Gly-DES, GO/Gly-DES) with an aqueous solution [54], graphene oxide nanosheets with aqueous solutions [57], polyacrylamide with an aqueous solution [57], carbon nanofibers with water [58], oxidized multi-walled carbon nanotubes wrapped by polyethylene glycol with water-based cutting fluid [59], and iron with Jatropha oil, are all of the nanoparticles with certain base fluids that are combined to form nanofluid. These nanofluids are later used in the drilling machining process, which helps in the reduction in the cutting temperature by providing an improvement in the surface in order to reduce thermal losses. In addition, it also helps to create a greater surface morphology with a smooth appearance, thus reducing the surface-roughness-like properties.

In the grinding machining operation, Al_2_O_3_ with emulsifier TRIM E709, nano-diamond with paraffin oil [68,69], diamond with deionized water [70], graphene nanoplates with IPA and TRIM SC200 [71], and carbon nanotubes with SAE-20W 40 oil all help to reduce the cutting temperature by controlling other thermophysical parameters and improving rheological properties. In the grinding machining operation, Al_2_O_3_ with water [72,76], Al_2_O_3_ with water [83], nano-diamond with paraffin oil, copper with water [81], and hBN with water soluble oil [88] all help to reduce the cutting temperature by controlling other thermophysical parameters and improving rheological properties.

In the process of turning, nano-graphite with water-soluble oil [90], GnP with vegetable oil [94], nano-boric acid with coconut oil [109,117], CNT, nano-boric acid with SAE40 oil [109], alumina with vegetable-based oil [110], MoS_2_ with vegetable oil [97], MWCNT [95], Al_2_O_3_ with vegetable oil [102], copper with vegetable oil [106], TiO_2_ with vegetable oil [97,98,119], MoS_2_ with coconut oil [107], i.e., different nanoparticles homogenized with different compatible base fluids, facilitate the overall process. These specifically mentioned nanofluids reduce the cutting surface temperature, thus avoiding thermal and heat losses and helping to maintain the balanced surface temperature. These balanced surface temperatures are helpful, i.e., they help in the maintenance of the morphology of the workpiece. As the carbon-based nanofluids have a perfect combination of physical, mechanical, and chemical properties that make them most suitable to be used in different machining operations, in a similar way, they have enhanced thermal properties related to heat transfer and thermal conductivity that make them the best-suited choice for the reduction in the cutting temperature.

## 4. Future Prospective and Conclusions

This paper outlined the use of the nanofluid as the major cutting (lubricating and/or cooling) fluid in certain machining operations, most notably in turning, drilling, milling, and grinding. The effect of different nanoparticles in the same machining operation was studied. Compared to the common fluids, these comparison studies generally show that nanofluids are more effective cutting fluids in machining operations. A few of the important observations that need to be highlighted are that they effectively reduce the surface roughness, cutting forces, and tool wear. Nanoparticles prevent the interaction between the workpiece and the tool and create some rolling mechanism that prevents the wear and tear caused by the friction. The nanofluids depict better tribological properties. Using these nanofluids improves the performance of the overall machining process, which is actually due to the improvement in the tool life and reduction in the cutting forces and cutting temperature, which lead to an increased overall performance of the specific machining operation.

Nanofluids have resulted in valuable research repute in various fields of research and development because of their enhanced properties, most importantly as the fluids for the heat transfer. Numerous studies have revealed the advantages, characteristics, limitations, and applicability of single-type nanoparticles and the applications of their colloidal mixtures, i.e., nanofluids. The discovery of the synergistic effect of nanoparticles, the part of the nanofluids relating to their improved thermophysical properties, has been further explored in experimental and theoretical studies of the hybrid nanofluids, while the major focus has been on thermal conductivity, viscosity, and specific heat capacity, which are the key thermal properties in the energy systems [124,125,126].

Until recent research, scientists have carried out nearly all possible methods regarding the application of a single nanofluid as the cutting fluid, whereas they can actually extend their research to hybrid nanofluids [127,128,129,130,131,132,133], which are more efficient in this application when compared to the single type of nanofluid. This is because hybrid nanofluids [134,135,136,137,138,139] show the maximum efficiency in the particular machining process among the different nanofluids. In addition, comparison studies in the branch of lubricating agents are mostly carried out based on the difference in the applications of nanofluids and ordinary fluids. However, in fact, the variation in different nanofluids (fluids of the same nature that just differentiate in the base material) would be more helpful if made as it would help to modify certain applications for the use and selection of a particular nanofluid; thus, the more the specification of the fluid and the base material matches, the greater the overall efficiency. Moreover, more in-depth studies and discussions on tribological evaluations by SEM, DLS, XPS, TEM, and AFM are strongly required to explore the physical mechanisms between the different nanoparticles and their relevant interfacial zones.

## Figures and Tables

**Table 1:** Nanofluids in milling machining operations.

Sr. No.	Nanoparticle Type	Base Fluid	Method of Lubrication	Workpiece Type	Tool Material	Findings/Improvements	References
**Carbon-Based Nanofluids**							
01.	Graphene sheets	Vegetable oil	MQL	Ti-6A1-4 V	Carbon steel Malleable iron	Flank wear decrease Central wear and cutting edge decreases Thermal conductivity enhancement by up to 1.038	[12]
02.	GnP	Vegetable oil	MQL	TC4 alloy	TiAIN-coated solid carbide end mill-tool	Reduces tool wear Reduces surface roughness Reduces cutting forces Reduces heat generation	[13]
03.	xGnP	Vegetable oil	MQL	AISI 1040	-	Lowers friction coefficient 7.45% decrease in tool wear Reduces cutting force 54.10% improvement in terms of surface finish	[13]
04.	Graphite nanoparticles	Vegetable-based cutting oil	MQL/NMQL	AISI 1040	Uncoated carbide tool	Tool life increase Decrease tool wear Decrease thermal conductivity by 50%	[14]
05.	Nanodiamond	Vegetable oil	MQL	Ti-6A1-4 V	Uncoated carbide twit drill	Decreases surface roughness	[15]
06.	GnP	Vegetable-based oil	MQL	Inconel X-750 superalloy	TiAIN-coated carbide	Improves surface roughness Decreases cutting forces Improvement in surface quality	[16]
07.	GO	Metalwork coolant	Conv	Ti6A14V	CBN tools (SECO)	Reduces friction forces Reduces cutting forces Uses 0.1 wt.% conc. of the nanoparticles	[17]
08.	Carbon onions	Alumicut oil	MQL	AL-2017-T4	Carbide tool	Cutting force reduced by 21.99% Surface roughness reduced by 46.32% Reduces coefficient of friction	[17]
09.	Graphite nanoplatelet	Distilled water	Wet	H-13 Steel	Uncoated micro-grain carbide	Cutting forces decrease Surface roughness Improves machining temperature Uses 1.6 wt.% conc. of the nanoparticles	[18]
10.	Nanocarbon onion	Mineral oil	MQL	AL-2017-T4	Carbide tool	Cutting force reduction by 21.99% Surface quality improvement by 46.32% It affects the appearance, function, and reliability of material	[19]
11.	GNP—SDS	Vegetable oil	MQL	Nickel-based super alloy Hastelloy X material	TiAIN-coated drills	Improves surface roughness Reduces cutting forces	[19]
**Metal-Based Nanofluids**							
12.	MoS_2_	Vegetable-based cutting oil	MQL/NMQL	AISI 1040	Uncoated carbide tool	Tool life increase Decreases tool wear Decreases thermal conductivity by 50%	[20]
13.	Fe_3_O_4_	Water and oil	Conv	Ti6A14V	Uncoated cemented carbide tools	Coefficient of friction reduced by 44.69% Contact angle reduced by 29.17% Flashpoint temperature decreased by 48.28%	[21]
14.	MoS_2_	Deionized water	MQL	Ti6A14V	Tungsten carbide flat end milling cutters	Surface wear reduced by 53.89% Provides better lubrication 7.15% reduced average temperature	[22]
15.	TiO_2_	Water	Conv	Mild steel	HSS end mill cutter	Decreases surface roughness Reduces cutting temperature	[23]
16.	MoS_2_	ECOCUT HSG 9055 oil	MQL	AL6061-T6	-	Surface roughness improves product quality Cutting temperature decrease by 56.8%	[24]
17.	MoS_2_	ECOCUT HSG 905S neat cutting oil	MQL	AI 6061-T6 alloy	Tungsten carbide	Cutting temperature minimized by adding 0.5 wt.% of the nanoparticle Surface roughness Cutting force	[25]
18.	MoS_2_	Vegetable-based oil	MQL	Inconel X-750 superalloy	TiAIN-coated carbide	Improves surface roughness Decreases cutting forces Improvement in surface quality	[26]
19.	TiO_2_	Cutting fluid	MQL	AISI 4340 steel	Carbide inserts with TiAIN coating	Improves surface roughness by up to 80% Improves spindle power Reduces wear of cutting inserts	[27]
20.	Copper nano-particle	Water-soluble oil	Wet	St 37	Carbide tool	Cutting temperature reduces cutting forces by 8.84% Surface roughness reduction by 14.74%	[28]
21.	Fe_3_O_4_	Conventional cutting fluid	Conv	Ti6A14V	Uncoated cemented carbide tools	Reduction in tool wear Reduction in adhesion of work piece Using 0.1 wt.% conc. of the nanoparticles	[29]
22.	TiO_2_	Lubricant emulsion (CLE)	Conv	16MnCr5 steel	Carbide milling inserts based on ISO:SNUN-120412	18.2% reduction in surface roughness Oxidation activation energy increased by 145%	[30]
23.	Cu	Oil	MQL	A17075-T6 aerospace alloy	Uncoated carbide mill cutter	Reduces surface roughness Reduces cutting forces by up to 84.66% Uses one wt.% of nanoparticles	[31]
24.	Ag, ZnO	EG	MQL	Inconel 718	Carbide inserts with TiAIN coating	Reduces surface roughness by 24.52% Reduces cutting temperature by 44.74% Improves surface finish by 13.07%	[32]
**Composite-Based Nanofluid**							
25.	TiO_2_	Lubricant emulsion (CLE)	Conv	16MnCr5 steel	Carbide milling inserts based on ISO:SNUN-120412	18.2% reduction in surface roughness Oxidation activation energy increased by 145%	[30,32]
26.	Montmorillonite clay	Water-soluble mineral oil	MQL	1080 steel	Carbide tool	Surface roughness Spindle load Cutting insert radius Uses conc. of 0.2–0.2 wt.%	[32]
27.	Al_2_O_3_	Water	Conv	Mild steel	HSS end mill cutter	6.27% reduction in average temperature Reduces surface roughness	[33]
28.	Al_2_O_3_	Oil	MQL	A17075-T6 aerospace alloy	Uncoated carbide mill cutter	Reduces surface roughness Reduces cutting forces by up to 84.66% Uses one wt.% of nanoparticles	[33]
29.	MMT clay	Water oil	Conv	AISI 1018 steel	WC/Co cemented carbides coated with TiAIN inserts	Surface roughness Minimum cutting forces	[26,34]
30.	SiO_2_	Mineral oil	MQL	A16061-T6	Carbide tool	Cutting temperature Volume friction Reduces cutting forces	[34,35]
31.	hBN	Vegetable-based oil	MQL	Inconel X-750 superalloy	TiAIN-coated carbide	Improves surface roughness Decreases cutting forces Improvement in surface quality	[34]
32.	hBN	Vegetable oil	MQL	Nickel-based super alloy Hastelloy X material	TiAIN-coated drills	Improves surface roughness Reduces cutting forces	[34]

**Table 4:** Nanofluids in turning machining operations.

Sr. No.	Nanoparticle Type	Base Fluid	Method of Lubrication	Workpiece Type	Tool Material	Findings/Improvement	References
**Carbon-Based Nanofluids**							
01.	MWCNT	Coconut oil	MQL	Martensitic Stainless Steel	Carbide tool insert	Cutting temperature Surface roughness	[90]
02.	Nano graphite	Water-soluble oil	MQL	AISI 1040	HSS/ cemented carbide tools	Reduces cutting temperature by 26% 3.1% reduction in surface roughness 65% reduction in cutting force 9% decrease in tool flank wear	[90]
03.	Graphite	Water-soluble oil	MQL	AISI 1040	HSS/ carbide tool	Cutting forces Average chip–tool interface temperature Tool wear Best surface quality at 2 wt.% MWCNTs nanofluid	[91]
04.	White graphite	Vegetable-based oil	MQL	AISI 1040 steel	Carbide tool	Surface roughness Tool wear High tool life 0.5% concentration	[92,93]
05.	GnP	Vegetable oil	MQL	AISI 1040	Coated carbide insert tool	Reduction in cutting temperature Reduction in cutting forces Reduction in surface roughness 10–15% Reduction in tool flank wear Improvement of tool life up to 10–15%	[94]
06.	MWCNT		MQL	AISI 1040	Multi-layered TiN top-coated insert	Cutting temperature Finite element analysis (FEAs)	[95]
07.	Carbon nanotubes (CNT)		MQL	AISI 1040	HS/ cemented carbide tools	29% reduction in cutting temperature Decrease surface roughness # by 34% 33% reduction in cutting force 39% decrease in tool wear	[96]
08.	GnP	Water-oil	MQL	AISI 304	Coated carbide insert Tool	Reduction in tool flank wear by up to 10–15% 10–15% tool life improvement Statistical significance on roughness	[96,97,98]
**Metal-Based Nanofluids**							
09.	MoS_2_ and TiO_2_	Vegetable oil	MQCL	Grade 23, Ti-6Al-4V ELI	Coated tungsten carbide (WC) cutting inserts	5.9% reduction in surface roughness Reduces cutting temperature by up to 45%	[97,99]
10.	AgNO_3_	Sodium borohydride	Wet	Mild steel	HSS	Cutting force Reduces wear scar diameter by 13% Surface roughness improvement 14% Tool temperature Improves load wear index by 8%	[99]
11.	CuO		MQL	AISI 1040	Cemented carbide insert-HSS	Workpiece cutting temperature reduction Improvement in tool life	[100]
12.	CuO	Water-soluble oil	Wet	AISI 4340	DNMG 150604-QM	Cutting forces Surface roughness Minimum quantity lubrication	[101]
13.	CuO	Coconut oil	MQL	AISI 1018	TiAlM-coated beyond blast insert	Reduces coefficient of friction by 53.89% Decreases wear track depth by 23.4% Decreases specific wear by 37.03%	[22,102]
14.	MoS_2_ Cu CuO	Grease	MQCL	Hardox 500 steel	Carbide tool	Surface quality Tool wear Weight friction of 10%	[103]
15.	ZnO, CuO, Al_2_O_3_	Deionized water	MQL	AISI 4340 steel	Uncoated cermet inserts	Improves surface finish Reduces tool wear Minimal tool vibration	[104]
16.	CuO	Deionized water	MQL	DSS-2205	Carbide-coated insert	Reduction in surface roughness Reduces cutting forces Decreases heat generation	[105]
17.	Cu	Vegetable oil	MQL	Bearing steel	Tungsten alloy make insert	60.65% reduction in surface roughness 11.13% minimization of cutting zone temperature	[106]
18.	Al_2_O_3_	Water-oil and magnetic field	Conv cooling	C45 steel	YT15 cemented carbide insert	Best stability at mass ratio of 5:1 Thermal conductivity Wettability	[98]
19.	MoS_2_	Coconut oil	MQL	AISI 1040	CNMG 120408 NC 6110 (coated carbide)	Cutting force Decreases cutting temperature by 22.6% Increases specific wear rate above 3% concentration	[107]
**Composite-Based Nanofluids**							
20.	Al_2_O_3_ and MWCNT	Deionized water and vegetable oil	MQL	AISI 304 steel	Coated cemented carbide insert	Surface roughness Machining force	[108]
21.	Boric acid solid lubricant	SAE-40 Coconut oil		AISI 1040 Steel	Carbide tool	Cutting temperature Surface roughness Tool wear 0.5% concentration	[109]
22.	Nano-boric acid	Coconut oil	Wet	AISI 304	Carbide tool (SNMG)	Cutting temperature Surface roughness Tool wear Increases thermal conductivity Increases coefficient of heat transfer	[109]
23.	Alumina	Vegetable-based oil	MQL	Inconel 625	Carbide tool	Recovers temperature by 58.32%	[110]
24.	hBN	Groundnut oil	MQL	Inconel 625	Coated cemented carbide insert	28.34% improvement for surface roughness 5.09% improvement for MRR	[110]
25.	Al_2_O_3_	Coconut Oil	MQL	AISI 1040	Coated carbide inserts	Curl diameter Lowers the temperature generated	[97]
26.	Al_2_O_3_	Vegetable oil	MQCL	Grade 23, Ti-6Al-4V ELI	Coated tungsten carbide (WC) cutting inserts	5.9% reduction in surface roughness Reduces cutting temperature by up to 45%	[97]
27.	CaF_2_ + MoS_2_	Vegetable oil	MQL	Hardened AISI H-13 steel	Tungsten carbide inserts	Depth of cut is 2 mm Cutting speed is 90 m/min Feed rate is 0.28 rev/min	[96]
28.	Al_2_O_3_	Coconut oil	MQL	AISI 1018	TiAlM-coated beyond blast insert	Reduces coefficient of friction by 53.89% Decreases wear track depth by 23.4% Decreases specific wear by 37.03%	[22,99]
29.	Al_2_O_3_	Vegetable oil	MQL	Inconel 600 alloy	Coated carbide cutting tool/uncoated carbide insert	Cutting temperature Surface roughness decreases by 0.3 µm for 1% vol. and 0.5 µm for 2% vol. Improved by approximately 25% more than pure MQL Tool wear	[102]
30.	SiO_2_	Mineral oil	MQL	AISI 4140 steel		Tool wear for 0.5% weight fraction	[111]
31.	Alumina	Coconut oil	MQL	AISI 1040 steel	Carbide tool	Best performance with 0.25% alumina nanoparticles	[103]
32.	Al_2_O_3_	Servo-Cut-‘S’	MQL	Ti-Ni alloy	SECO Roughing coated carbide insert	Tool wear Reduces cutting force by up to 30 to 50% 54.5 and 28.5% reduction in surface roughness Chip thickness	[112]
33.	Al_2_O_3_	Water	nMQL	Nickel-based Nimonic 90 alloy	AlTiN-coated tungsten carbide inserts	High productivity Good surface quality 22–25% improvement in surface finish	[113]
34.	Al_2_O_3_, hBN	DW-oil	MQCL	Inconel 625	Tungsten carbide insert	37% reduction in tool wear 44% reduction in surface roughness 0.5% vol.	[114]
35.	Al_2_O_3_	Deionized water	MQL	AISI 4340 steel	Uncoated cermet inserts	Improves surface finish Reduces tool wear Minimal tool vibration	[104]
36.	Al_2_O_3_	Vegetable oil and water	MQL	Inconel 718	Uncoated carbide inserts	Helix angles of chips increase with decreasing cutting speed	[105]
37.	Al_2_O_3_	Deionized water	MQL	DSS-2205	Carbide-coated insert	Reduces surface roughness Reduces cutting forces Decreases heat generation	[105]
38.	Al—GnP	Water-oil	MQL	AISI52100 steel	Uncoated carbide inserts (YG8)	Improves thermal conductivity by 3.48% for 0.2% vol., 7.44% for 0.7% vol., 9.03% for 1.2% vol. Increases viscosity by 17.21% for 0.20% conc., 23.54% for 0.75% conc., 39.24% for 1.20% conc.	[115]

## Data Availability

Not applicable.
